# Chirobiophore: A Novel Framework for Quantifying Biochirality in Macromolecular Systems

**DOI:** 10.3390/biom16040576

**Published:** 2026-04-13

**Authors:** Claudiu N. Lungu, Subhash C. Basak

**Affiliations:** 1 Department of Functional and Morphological Science, Faculty of Medicine and Pharmacy, Dunarea de Jos University, 8000080 Galati, Romania; 2 Department of Chemistry and Biochemistry, University of Minnesota, 246 Chemistry Building, 1039 University Drive, Duluth, MN 55812, USA; sbasak@d.umn.edu

**Keywords:** biochirality, membrane proteins, structural asymmetry, chirality descriptors, protein topology, molecular geometry, topological analysis, vector field modeling, protein design, morphogenetic patterning, chirobiophore

## Abstract

Chirality is a pervasive and functionally critical feature of biological macromolecules, yet its distributed and emergent forms remain poorly quantified in complex systems such as membrane proteins. We present Chirobiophore, a novel paradigm for capturing biochirality across scales—from atomic geometries to global structural asymmetries. Unlike traditional stereochemical metrics, Chirobiophore employs a multidimensional model-independent vector comprising Local Tetrahedral Asymmetry (LTA), Helical Path Curvature (HPC), Asymmetric Environment Score (AES), Directional Density Profile (DDP), Leaflet Asymmetry Index (LAI), and Orientation Twist Score (OTS). This framework enables coordinate-invariant comparisons of structurally diverse proteins in a continuous chirality space. We demonstrate its application to canonical, GPCR, and topologically complex membrane proteins, revealing distinct chirality signatures and functional clustering. Furthermore, we map Chirobiophore descriptors to tissue-level asymmetry indices, providing a bridge between molecular structure and morphogenetic patterning. Chirobiophore offers a unified, extensible platform for structural biology, synthetic design, and developmental modeling of chirality.

## 1. Introduction

Biological systems exhibit chirality at every scale: amino acids are L-enantiomers, alpha-helices are right-handed, and membrane proteins often insert asymmetrically. Traditional chirality metrics capture stereocenters but fail to quantify emergent or distributed chirality in complex and multiscale structures. We define a new paradigm of biochirality that operates independently of model orientation and sequence labels, focusing on intrinsic geometry and structural motifs [1].

Chirality—handedness—is a fundamental property of biological systems, governing molecular recognition, enzymatic catalysis, and supramolecular assembly. From the stereospecificity of amino acids and sugars to the asymmetric architecture of proteins, nucleic acids, and membranes, biological function is intimately tied to chirality at every scale. At the molecular level, life exhibits a striking homochirality: proteins are constructed almost exclusively from L-amino acids, while nucleic acids use D-ribose or D-deoxyribose as their sugar backbones. These foundational asymmetries are amplified in higher-order structures: α-helices are predominantly right-handed in natural proteins, DNA helices are predominantly right-handed B-form (and occasionally left-handed Z-form under specific conditions), and even cellular membranes exhibit asymmetric insertion and lateral distribution of proteins and lipids. Chirality thus pervades the entire structural hierarchy of biology, from biologically important macromolecules to building blocks such as amino acids and sugars, for the whole organism [2,3].

However, despite its centrality, chirality remains poorly quantified in complex biological systems. Traditional chirality metrics—such as stereocenter configuration (R/S), helical sense, and scalar measures like the Hausdorff chirality measure or chiral volume—are typically local and continuous. They excel in small-molecule chemistry, where individual stereocenters determine function and identity. Yet they fall short when applied to macromolecules and supramolecular assemblies where chirality is often distributed, emergent, and topologically encoded. In such cases, chirality is not reducible to individual atoms or residues but arises from the spatial arrangement of motifs across multiple scales [4,5].

To address this conceptual and methodological gap, we propose a new paradigm, the chirobiophore. Drawing inspiration from the well-established concept of the pharmacophore, first proposed by Kier in medicinal chemistry, a chirobiophore is defined as the ensemble of spatial features within a biological structure that quantifies its degree of handedness. Just as a pharmacophore abstracts the minimal steric and electronic requirements for binding to a biological target, a chirobiophore abstracts the minimal geometric and topological requirements that give rise to functional chirality in a system; it is not limited to local stereocenters or specific chemical groups. Instead, it captures the emergent handedness that governs interactions, folding, assembly, and recognition [6].

This analogy with pharmacophores is more than rhetorical; it is methodologically productive. In drug design, a pharmacophore enables researchers to search for or generate molecules that match a known binding geometry, regardless of the detailed molecular architecture. Similarly, a chirobiophore could allow researchers to classify and compare structures based on their chiral geometry, even when the underlying sequences or detailed three-dimensional structures differ from one another. This could enable, for instance, the identification of conserved chiral motifs across divergent protein folds, or the discovery of functionally convergent chirality in synthetic peptides, nucleic acids, or nanostructures [7,8].

A pharmacophore is typically described in terms of features such as hydrogen bond donors/acceptors, hydrophobic centers, and charged groups, along with their three-dimensional spatial relationships. In the chirobiophore framework, features may include helical handedness, curvature, torsional waveforms, asymmetric motif packing, or vector fields derived from backbone traces. These features can be encoded using mathematical tools such as differential geometry, topology, persistent homology, or local vector chirality measures. Importantly, the chirobiophore is coordinate-invariant (independent of overall orientation or labeling), scale-flexible, and applicable to dynamic ensembles, such as those derived from molecular dynamics or cryo-EM reconstructions [7,9].

Chirobiophores can be identified and compared using computational methods, such as those developed for shape matching, graph comparison, and spatial motif analysis. One promising direction is the use of geometric deep learning to extract chiral patterns from structural data. In analogy with how convolutional neural networks identify pharmacophoric patterns in chemical graphs or grids, geometric networks operating on point clouds or meshes can learn chiral descriptors that generalize across structures and contexts. Alternatively, descriptors based on curved manifolds, vector chirality indices, or frame-invariant tensor representations offer analytic routes to quantify handedness without relying on sequence or symmetry priors [10].

The biological implications of chirobiophores are far-reaching. In protein design, chirobiophores can help enforce or assess the desired handedness in designed folds, eliminating the need to encode chirality at every level explicitly. In evolutionary biology, comparing chirobiophores across homologous proteins may reveal conserved asymmetries that are otherwise obscured by sequence divergence. In pathology, aberrant chirobiophore patterns may serve as signatures of misfolding or aggregation, particularly in amyloid diseases where chirality shifts accompany phase transitions. In synthetic biology, modular chirobiophores can inform the design of chiral scaffolds, foldamers, or nanomachines with tailored mechanical or optical properties [11].

Beyond biology, chirobiophores offer a bridge to other chiral systems in chemistry and materials science. For example, chiral metamaterials, liquid crystals, and self-assembled helices exhibit emergent handedness that chirobiophoric descriptors may more appropriately describe than by simple stereochemical labels. Just as pharmacophores provided a new paradigm in rational drug design, chirobiophores could enable a structure–function framework for chirality in both natural and synthetic systems [12].

Moreover, the chirobiophore concept provides a unifying vocabulary across disciplines that have traditionally treated chirality in fragmented ways. It links the discrete chirality of stereocenters with the continuous chirality of twisted filaments and the topological chirality of knots and braids. It encourages the development of chirality-aware tools and databases, which can annotate biomolecular structures not only by secondary structure or domain type, but also by their chirobiophoric signature—capturing an essential but underexplored axis or attribute of biological identity [13].

In conclusion, the chirobiophore represents a novel foundational advancement in the way we define, detect, and utilize chirality in biological and complex chemical systems. Shifting the focus from isolated stereocenters to distributed, emergent handedness opens new avenues for structural analysis, functional interpretation, and rational design. Like the pharmacophore before it, the chirobiophore promises to be a powerful abstraction—one that will illuminate the hidden geometry of life [14].

This study aims to develop and validate a comprehensive framework—termed *Chirobiophore*—for quantifying structural chirality in macromolecular systems, particularly membrane proteins, using multiscale, coordinate-invariant descriptors. This framework aims to bridge atomic-level stereochemistry with global asymmetry patterns present in complex biological systems in a unified geometric space [15].

We hypothesize that Chirobiophore descriptors can distinguish between protein classes with different structural and functional roles based on their chirality signatures, and that these signatures can be meaningfully mapped onto tissue-level asymmetry indices. This implies that chirality is not only a molecular attribute but also a translatable feature across biological scales [16].

To address this conceptual and methodological gap, we propose a new class of structural descriptors: the chirobiophore. Drawing inspiration from the well-established concept of the pharmacophore in medicinal chemistry, a chirobiophore is defined as the ensemble of spatial features within a biological structure that collectively quantifies its intrinsic chiral geometry [17].

The primary goal of this work is to introduce and formalize the *Chirobiophore* concept as a computational and geometric framework for quantifying distributed chirality in macromolecular systems. The present study is intended as a proof-of-concept demonstration rather than a comprehensive validation across all membrane protein classes.

Accordingly, the emphasis of this manuscript is on (i) defining mathematically well-posed chirality descriptors, (ii) demonstrating their coordinate-invariant behavior, and (iii) illustrating their potential discriminatory power on a representative but limited set of membrane protein structures. While selected biological interpretations and multiscale extensions are discussed, these should be understood as hypothesis-generating perspectives intended to motivate future experimental and computational work. As stated before this work is intended as a proof-of-concept framework that defines and demonstrates a multiscale, coordinate-invariant representation of distributed chirality, rather than as a fully validated predictive model. Membrane proteins are selected as a representative testbed because they exhibit inherently multiscale chirality coupled with strong spatial asymmetry due to bilayer embedding, making them particularly suitable for demonstrating the capabilities of the framework.

## 2. Materials and Methods

To validate the Chirobiophore framework described in the manuscript (also Appendix A), a representative set of structurally diverse membrane proteins from the Protein Data Bank (PDB) that span a range of chiral and topological features can be used. Here’s a proposed molecular system and PDB selection strategy to systematically compute and compare all Chirobiophore descriptors:

To validate the Chirobiophore descriptor system and ensure its applicability across a broad spectrum of membrane protein architectures, we propose a carefully curated molecular system based on publicly available structures from the Protein Data Bank (PDB). The selected proteins are grouped into three categories, each representing a different level of chirality complexity. This stratification allows for a comprehensive assessment of the framework’s sensitivity to local, global, and membrane-embedded structural asymmetries.

The first group consists of canonical α-helical membrane proteins, including bacteriorhodopsin (PDB: 1C3W) [18], aquaporin-1 (PDB: 1J4N) [19], and rhodopsin (PDB: 1F88) [20]. These proteins are characterized by their predominantly helical and symmetric architectures, making them ideal for benchmarking fundamental descriptors such as Local Tetrahedral Asymmetry (LTA), Helical Path Curvature (HPC), and Orientation Twist Score (OTS). Their regular geometry ensures a controlled environment for isolating chirality features associated with secondary structure coherence and minimal embedding complexity.

The second group targets G protein-coupled receptors (GPCRs) and other asymmetric membrane proteins that display complex folding patterns and pronounced directional embedding. Structures such as the β2-adrenergic receptor (PDB: 2RH1) [21], the μ-opioid receptor (PDB: 4DKL) [22], and the chemokine receptor CXCR4 (PDB: 3ODU) [23] offer a rich testing ground for descriptors sensitive to asymmetric packing and membrane insertion patterns. These proteins are particularly valuable for evaluating the Asymmetric Environment Score (AES), Depth Distribution Profile (DDP), and Leaflet Asymmetry Index (LAI), as they often exhibit distinct biases in their cytoplasmic and extracellular domains, linked to their signaling roles.

The third group comprises structurally irregular or topologically complex membrane proteins that challenge conventional symmetry and packing assumptions. This set includes the voltage-gated potassium channel (PDB: 2A79) [24], the ATP synthase subunit c (PDB: 1C17) [25], and the ClC chloride transporter (PDB: 1OTS) [26]. These proteins feature mixed secondary structures, twisted domains, and unconventional embeddings, serving as robust test cases for stress-testing the generalizability and robustness of the Chirobiophore descriptors under atypical conditions.

Protein grouping in this study is based primarily on structural characteristics rather than functional classification. Canonical α-helical membrane proteins include structures with well-defined transmembrane helices, among which GPCRs represent a specialized functional subclass. Accordingly, rhodopsin is structurally categorized within the α-helical group while also belonging to the GPCR family.

**Figure 1:**
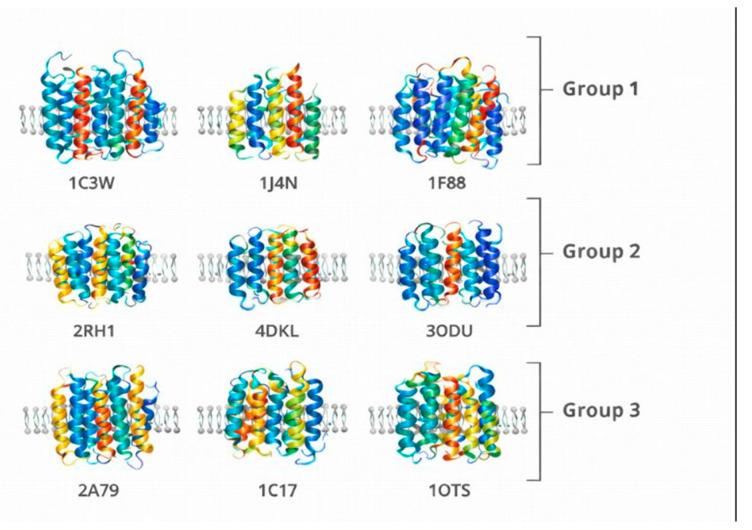
Representative membrane protein structures analyzed in this study. Ribbon representations of the nine membrane proteins analyzed in this study, grouped into canonical α-helical proteins (Group 1), GPCRs and asymmetric membrane proteins (Group 2), and structurally complex membrane proteins (Group 3). Structures are shown in membrane-aligned orientation to illustrate architectural diversity relevant to the Chirobiophore analysis.

Each protein will be analyzed by computing its full Chirobiophore vector, comprising LTA, HPC, AES, DDP, LAI, and OTS, after alignment along a consistent membrane-normal axis (typically Z). The resulting vectors will be projected into lower-dimensional space using techniques such as principal component analysis (PCA) or t-distributed stochastic neighbor embedding (t-SNE) to visualize clustering behavior. This analysis will determine whether proteins with similar chirality profiles cluster by structural class, functional role, or evolutionary lineage (Figure 1) (Supplementary Materials File S1).

Optionally, selected proteins may also be subjected to molecular dynamics (MD) simulations to evaluate the temporal stability and variability of Chirobiophore vectors over time. This dynamic validation could reveal trajectory patterns, conformational switches, or topological transitions within chirality space, further underscoring the biological relevance of the descriptors.

Through this structured and biologically diverse benchmark, we aim to demonstrate the utility, interpretability, and discriminatory power of the Chirobiophore framework across a range of real-world macromolecular systems.

### 2.1. Computational Overview

All Chirobiophore descriptors are computed directly from atomic coordinates extracted from Protein Data Bank (PDB) structures using a standardized, coordinate-invariant workflow. Briefly, each structure undergoes (i) preprocessing and alignment relative to a membrane reference frame, (ii) descriptor-specific geometric analysis at local, mesoscopic, and global scales, and (iii) normalization to ensure comparability across structures of different sizes and topologies. Structural data were obtained from the Protein Data Bank (PDB) and processed using standard coordinate formats. The computational pipeline is compatible with both legacy PDB and mmCIF formats, depending on data availability. Atomic coordinates and connectivity information were extracted using format-independent parsing procedures to ensure consistency and reproducibility across different structure file types. Helix boundaries were obtained directly from Protein Data Bank (PDB) annotations (HELIX records). These annotated segments were used to define helix regions for subsequent axis computation and descriptor analysis. This approach ensures consistency with experimentally curated structural definitions while avoiding variability introduced by secondary structure assignment algorithms. Alternative definitions (e.g., DSSP-based assignments) may be explored in future work.

Distances between Chirobiophore vectors were computed using the Euclidean distance in standardized descriptor space. Each descriptor was z-score normalized to ensure comparability across different scales. For two proteins with descriptor vectors C_1_ and C_2_, the distance was defined as:

d(C_1_, C_2_) = √∑_i_ (C_1i_ − C_2i_)^2^

This metric allows balanced contribution of all descriptors and provides a basis for clustering and similarity analysis.

The complete computational pipeline follows the sequence: PDB preprocessing → membrane alignment → descriptor computation → normalization → vector assembly, resulting in a six-dimensional Chirobiophore vector C = [LTA, HPC, AES, DDP, LAI, OTS]

All steps are deterministic and independent of residue labeling, atom naming conventions, or global coordinate orientation.

### 2.2. Structure Preprocessing and Coordinate Alignment

Prior to descriptor computation, all structures were stripped of solvent molecules and non-protein heteroatoms unless explicitly required for a given descriptor. Atomic coordinates were translated so that the protein center of mass coincided with the origin.

For membrane proteins, a membrane reference frame was defined by aligning the principal axis of transmembrane helices with the global *z*-axis, corresponding to the membrane normal. When available, experimentally annotated membrane orientations were used; otherwise, the transmembrane axis was inferred using helix principal component analysis.

All subsequent descriptor calculations are invariant under rigid-body rotation and translation (Algorithms 1–7). Hydrogen atoms were not included in the LTA calculation, as most Protein Data Bank (PDB) structures analyzed in this study do not contain experimentally resolved hydrogen coordinates. To ensure reproducibility and avoid model-dependent bias, LTA was computed using heavy atoms only. Tetrahedral configurations were constructed from atoms with sufficient bonded neighbors, and atoms lacking the required coordination were excluded from the calculation. Only atoms with sufficient bonded neighbors to define a valid tetrahedral configuration were included in the LTA calculation. Specifically, tetrahedra were constructed from a central atom and three neighboring atoms. Atoms lacking the required coordination (e.g., terminal atoms or atoms with fewer than three bonded neighbors) were excluded to ensure geometric consistency of the descriptor.

Helices containing proline residues, which may introduce local kinks or distortions, were handled naturally within the PCA-based framework. The global helix axis, computed from all Cα atoms in the helix, reflects the dominant overall direction and is therefore robust to localized deviations. In contrast, the sliding window PCA used for local axis estimation captures such kinks explicitly as local changes in orientation. This allows proline-induced distortions to be incorporated into downstream descriptors that quantify torsional and orientational variability.

A fixed cutoff radius of 6 Å was used to define local atomic neighborhoods for AES computation. This value was selected as a compromise between capturing sufficiently local structural environments and maintaining statistical robustness across diverse protein architectures. While atom-type-dependent or adaptive cutoffs could provide a more detailed representation of chemical environments, a uniform cutoff was adopted here to ensure consistency and comparability of the descriptor across all analyzed structures. Exploration of adaptive or chemically informed cutoff schemes represents a potential direction for future refinement.

The following algorithm was used:
**Algorithm 1:** Chirobiophore Descriptor PipelineInput: PDB structure Output: Chirobiophore vector C = [LTA, HPC, AES, DDP, LAI, OTS]  Load atomic coordinates from PDB file  Remove solvent and optional heteroatoms  Align structure to membrane reference frame (*z*-axis = membrane normal)  For each descriptor did_idi:  a. Compute raw geometric measure  b. Apply descriptor-specific normalization  Assemble normalized values into vector CCC  (Optional) Standardize CCC for multivariate analysis
**Algorithm 2:** LTA ComputationInput: Atomic coordinates
Identify valid tetrahedra (central atom + 3 neighbors)Compute signed tetrahedral volumesAverage signed volumes over structureNormalize by mean absolute volume
**Algorithm 3:** HPC ComputationInput: Cα backbone coordinates
Extract contiguous four-residue backbone segmentsCompute torsion angle for each segmentAssign sign based on handednessAverage torsion values over all segments
**Algorithm 4:** AES ComputationInput: Atomic coordinates Define local neighborhood for each atom (cutoff radius)Construct angular distribution of neighborsCompute Shannon entropy of angular histogramAverage entropy values across atoms
**Algorithm 5:** DDP ComputationInput: Atomic coordinates aligned to membrane normal
Bin atoms along z-axisCompute normalized density profileCalculate skewness of density distribution
**Algorithm 6:** LAI ComputationInput: Atomic coordinates, membrane midplane
Partition atoms into upper and lower half-spacesCount atoms (or weighted chirality values) per leafletCompute normalized difference between leaflets
**Algorithm 7:** OTS ComputationInput: Transmembrane helix axes
Compute helix axis vectorsMeasure tilt angles relative to membrane normalAssign handedness from backbone torsionCompute signed average tilt

### 2.3. Chirobiophore Descriptor Definitions

Each Chirobiophore descriptor captures a distinct geometric manifestation of chirality and is computed using explicitly defined mathematical operations and parameters, as summarized below. Unless otherwise stated, all descriptors are normalized to yield dimensionless quantities suitable for cross-structure comparison.

LTA values were normalized by the mean absolute tetrahedral volume computed across the structure, ensuring scale invariance with respect to protein size and resolution. HPC was computed over contiguous backbone segments using Cα atoms only, with torsion values averaged over all valid four-point paths; the final score represents the signed mean torsional curvature per residue. For AES computation, angular space was discretized into uniform bins (Δθ = 15°), and Shannon entropy was calculated for each atomic neighborhood within a fixed cutoff radius of 6 Å. Density profiles were normalized by total atom count prior to skewness calculation, ensuring comparability between structures of different sizes. When explicit lipid atoms were not present, LAI was computed using protein atom distributions relative to the membrane midplane, with this limitation explicitly acknowledged in the interpretation. OTS was computed as the signed mean tilt angle of transmembrane helices relative to the membrane normal, with handedness determined from backbone torsion sign conventions.

To allow direct comparison between structures, all descriptor values were normalized either by intrinsic geometric scales (e.g., mean volume, atom count) or by statistical standardization across the dataset. The resulting dimensionless descriptors were assembled into the final Chirobiophore vector without additional weighting unless explicitly stated.

### 2.4. Reproducibility and Data Availability

All Chirobiophore descriptors reported in this study are computed using deterministic algorithms applied directly to atomic coordinates obtained from publicly available Protein Data Bank (PDB) structures. To ensure full reproducibility, all scripts used for structure preprocessing, membrane alignment, descriptor computation, normalization, and figure generation will be made publicly available in an open access repository upon revision.

The repository will include source code, input structure lists (PDB IDs), parameter files, example execution workflows, and reference outputs corresponding to the results reported in this manuscript.

The set of membrane protein structures analyzed in this study was intentionally selected to span multiple architectural and functional classes, including canonical α-helical membrane proteins, G protein-coupled receptors, and topologically complex transporters and channels. This selection was designed to provide structural diversity rather than statistical completeness, allowing us to assess whether the proposed descriptors respond meaningfully to qualitatively different forms of macromolecular chirality.

A primary limitation of the present study is the relatively small number of analyzed structures. As a result, the observed clustering patterns and descriptor distributions should not be interpreted as statistically definitive classifications of membrane protein chirality. Instead, they demonstrate the feasibility and discriminatory potential of the Chirobiophore framework when applied to structurally diverse examples.

Comprehensive validation across larger datasets will be required to establish population-level trends, confidence intervals, and class-specific thresholds for individual descriptors.

The dataset is not intended to exhaustively sample membrane protein space, but rather to serve as a representative proof-of-concept benchmark for evaluating descriptor behavior across distinct structural regimes.

From a computational perspective, the Chirobiophore framework is inherently scalable, as each descriptor is computed independently from atomic coordinates with linear or near-linear complexity in system size. Extension to larger datasets or ensemble-based analyses therefore requires no modification of the underlying methodology.

All computational steps follow a modular workflow in which individual descriptors can be computed independently or jointly assembled into a Chirobiophore vector. This modularity enables straightforward reproduction of individual results and facilitates future extension of the framework to additional descriptors or datasets.

The present study focuses on deterministic descriptor values computed from individual static protein structures and does not yet incorporate ensemble-based variability, experimental uncertainty, or conformational sampling. As such, reported Chirobiophore values should be interpreted as structure-specific descriptors, not as population means or statistically converged estimates.

Descriptor comparisons presented in this section reflect relative differences between proteins within the analyzed dataset and are intended to illustrate trends rather than establish statistically significant separations between protein classes. No assumptions of normality or class-level distributions are made at this stage.

Sensitivity of the Chirobiophore descriptors to coordinate alignment, binning resolution, and neighborhood cutoffs was qualitatively assessed to ensure numerical stability. Descriptor definitions were chosen to minimize dependence on arbitrary parameter choices, and future work will include systematic sensitivity analyses to quantify parameter-induced variance.

Because the present analysis is based on a limited number of static structures, the study does not yet provide confidence intervals, variance estimates, or hypothesis tests for individual descriptors. Consequently, observed clustering patterns and gradients should be interpreted as qualitative indicators of descriptor behavior rather than statistically validated separations. Establishing statistical robustness will require larger datasets and ensemble-based analyses.

Future extensions of the Chirobiophore framework will incorporate statistical characterization of descriptor distributions across protein families, uncertainty estimates derived from conformational ensembles (e.g., molecular dynamics trajectories), and formal hypothesis testing where appropriate. These developments will enable rigorous assessment of descriptor robustness and biological relevance.

### 2.5. Descriptor Definitions

To capture the distributed and emergent nature of chirality in biological macromolecules, particularly membrane proteins, we introduce a suite of geometric descriptors. These descriptors form the components of the Chirobiophore vector, a multiscale, orientation-invariant representation of structural asymmetry. Each descriptor targets a specific scale or mode of chirality, from local atomic arrangements to global membrane-spanning geometry.

We begin by formally defining three core descriptors: Local Tetrahedral Asymmetry (LTA), Helical Path Curvature (HPC), and the Asymmetric Environment Score (AES). Each provides unique geometric insight into different layers of chiral structure.

Local Tetrahedral Asymmetry (LTA)

Purpose:

LTA quantifies the local handedness of atomic arrangements by evaluating the signed volume of tetrahedra formed by a central atom and three of its bonded neighbors. This descriptor captures chirality at the finest structural scale—atomic geometry.

Mathematical Definition:

Given four atoms with positions *ri*, *rj*, *rk*, *rl*, the signed volume of the tetrahedron they form is:

$$V_{ijkl}=\frac{1}{6}\cdot \left[\left(rj-ri\right)\cdot \left(\left(rk-ri\right)\times \left(rl-ri\right)\right)\right]$$

The sign of this volume indicates the handedness (left or right) of the local configuration. LTA is then computed as the normalized average of signed volumes across all valid tetrahedra in the system:

$$LTA=\frac{1}{N}\sum_{n=1}^{N}sign\left(V_{n}\right)\cdot \left|\frac{V_{n}}{V}\right|$$

where V^−^ is a normalization factor (e.g., mean absolute volume) and N is the total number of tetrahedra considered.

Helical Path Curvature (HPC)

Purpose:

HPC assesses backbone-level chirality by quantifying the signed torsion angles along sequential atomic segments. This descriptor reflects the handedness and torsional coherence of the backbone, rather than curvature in the strict differential geometric sense. The sign of the torsion encodes local handedness, while its consistency across the structure captures global helical organization.

Mathematical Definition:

For a four-atom segment with positions r⃗ 1, r⃗ 2, r⃗ 3, r⃗ 4 define:

b⃗ _1_ = r⃗ _2_ − r⃗ _1_

b⃗ _2_ = r⃗ _3_ − r⃗ _2_

b⃗ _3_ = r⃗ _4_ − r⃗ _3_

From these, compute two normal vectors:

n⃗ _1_ = b⃗ _1_ × b⃗ _2_

n⃗ _2_ = b⃗ _2_ × b⃗ _3_

Then, the torsion angle θ is:

θ = arctan2(‖b⃗ _2_‖·(b⃗ _1_·n⃗ _2_), n⃗ _1_·n⃗ _2_)

The sign of θ determines the local handedness of that segment. The overall descriptor is computed as:

$$HPC=\frac{1}{M}\sum_{I=l}^{M}sign(\theta_{i})$$

where

*M* is the number of four-atom paths (typically along backbone or helical axes).

Asymmetric Environment Score (AES)

Purpose:

AES captures the degree of spatial asymmetry in the local environment around each atom. It is beneficial for detecting irregular or functionally diverse regions, such as protein–lipid interfaces or disordered loops. Mathematical Definition: For a given atom *i*, the AES is derived from the angular distribution of its neighboring atoms. This distribution is converted into a probability histogram over angular bins k, producing normalized values *p_ik_*:

$$p_{ik}=\frac{H_{ik}}{\sum_{k}H_{ik}}$$

where *H_ik_* is the number of neighbors in bin *k*, the local entropy is then:

$$S_{i}=-\sum_{k}p_{ik}logp_{ik}$$

The final AES is the average entropy across all atoms:

$$AES=\frac{1}{N}\sum_{i=1}^{N}S_{i}$$

Low entropy implies highly symmetric local environments; high entropy reflects asymmetric or heterogeneous packing. For each atom, neighboring atoms within a cutoff radius of 6 Å are identified, and their positions are expressed in a local spherical coordinate system centered on the atom. The angular distribution of neighbors is discretized using uniform binning of the polar angle (θ) and azimuthal angle (φ), forming a two-dimensional angular histogram over the unit sphere.

The histogram is normalized to obtain a probability distribution over angular bins, and the Shannon entropy of this distribution is computed to quantify the degree of spatial asymmetry in the local environment. Lower entropy values correspond to more symmetric, structured environments, whereas higher entropy values indicate more heterogeneous or asymmetric spatial distributions.

Directional Density Profile (DDP)

Purpose:

DDP characterizes the anisotropic distribution of atomic density along a given axis, typically aligned with the membrane normal. The Directional Density Profile (DDP) is derived from the spatial distribution of atomic density along the membrane normal. While the underlying quantity is a full density profile constructed from binned atomic positions, the descriptor reported here is a scalar summary statistic—specifically, the skewness of this distribution. Thus, the term “profile” refers to the underlying distribution rather than the final scalar value used for analysis.

This descriptor is especially suited for membrane proteins, where asymmetry often emerges along the direction perpendicular to the bilayer plane. Mathematical Definition: Let z denote the coordinate along the chosen axis (e.g., membrane normal). The spatial domain is partitioned into a set of evenly spaced slabs (or bins) along zzz. For each bin bbb, count the number of atoms nbn_bnb residing within it to generate a density profile ρ the DDP is then computed as a skewness metric of this distribution:

$$DDP=\frac{1}{\sigma^{3}\;N}\sum_{b=1}^{N}{{(z}_{b-\mu })}^{3}\cdot n_{b}$$

where:

*μ* is the mean position of the density distribution;

*σ* is the standard deviation;

*N* is the total number of bins;

*n_b_* is the atom count in bin *b*;

*z_b_* is the central coordinate of bin *b*.

A nonzero skewness indicates an asymmetric distribution of matter along the axis, consistent with chiral asymmetry. The skewness metric inherently assigns greater weight to atoms located farther from the mean due to the cubic dependence on deviations. This property is not arbitrary but reflects the sensitivity of skewness to directional asymmetry in the distribution. In this context, atoms that are more strongly displaced along the membrane normal contribute more prominently to the descriptor, capturing structural bias in embedding and spatial organization.

Lipid Asymmetry Index (LAI)

Purpose: LAI is designed to detect asymmetry in the composition and arrangement of lipids around membrane proteins. Since proteins often interact unevenly with the inner vs. outer leaflets of the membrane, LAI provides a scalar summary of this bias.

In the present study, the Leaflet Asymmetry Index (LAI) was computed without explicit lipid bilayers, as most analyzed Protein Data Bank structures do not contain fully resolved membrane environments. Instead, LAI was derived from the spatial distribution of protein atoms relative to an inferred membrane midplane.

Specifically, following alignment of the protein to a membrane reference frame, the structure was partitioned into two half-spaces along the membrane normal (*z* = 0). Protein atoms (or residue centroids) located at *z* > 0 were assigned to the upper leaflet proxy, while those at *z* < 0 were assigned to the lower leaflet proxy. LAI was then computed as the normalized difference in atom counts between these two regions. While this formulation does not measure lipid compositional asymmetry, it captures protein embedding asymmetry with respect to the membrane plane. As such, LAI should be interpreted as a descriptor of structural leaflet bias of the protein itself, rather than as a direct measure of bilayer lipid asymmetry. Because explicit lipid bilayers were not included, LAI values reported here do not reflect lipid–protein compositional asymmetry or leaflet-specific lipid interactions. Consequently, LAI should be interpreted as a geometric proxy for protein orientation and depth bias within the membrane rather than as a direct measurement of membrane asymmetry. Future implementations of LAI may incorporate explicit lipid coordinates derived from co-crystallized structures, membrane modeling tools, or molecular dynamics simulations, enabling direct quantification of lipid–protein leaflet asymmetry. Such extensions would allow separation of protein-intrinsic embedding bias from bilayer compositional effects.

Mathematical Definition: Consider two regions flanking the membrane midplane—typically corresponding to the cytoplasmic and extracellular (or periplasmic) leaflets. Let *N_upper_* and *N_lower_* represent the number of lipid atoms, headgroups, or residues in the respective regions within a fixed radius of the protein. The LAI is defined as:

$$LAI=\frac{N_{upper}-N_{lower}}{N_{upper}+N_{lower}}$$

Values range from −1 to 1:

LAI = 0 implies perfect symmetry;

LAI > 0 implies more lipids in the upper region;

LAI < 0 implies more in the lower region.

This descriptor can also be generalized to compare specific lipid species, charge distributions, or interaction energies.

Orientation Tensor Skew (OTS)

Purpose: OTS measures the geometric asymmetry of the mass or shape distribution by analyzing the skew of the orientation (or inertia) tensor. It captures the global handedness of a 3D object beyond specific local features. The Orientation Twist Score (OTS) is defined as a signed measure combining helix orientation relative to the membrane normal with torsional handedness derived from backbone geometry. It reflects both the directional alignment of helices and their rotational sense, providing a global descriptor of membrane-spanning chirality.

Mathematical Definition: Construct the orientation tensor T based on all atomic positions ri, typically centered on the center of mass:

$$T_{\alpha \beta }=\sum_{i=l}^{N}\omega_{i}\left(ri\alpha -r^{ˉ}\;\alpha \right)\cdot \left(ri\beta -r^{ˉ}\;\beta \right)$$

where:

*α*,*β* ∈ {x,y,z};

wi is a weighting factor (e.g., atomic mass or uniform);

rˉα is the center-of-mass coordinate along axis *α*.

Compute the eigenvalues *λ*1, *λ*2, *λ*3 of T, ordered such that *λ*1 ≤ *λ*2 ≤ *λ*3. The skew of this spectrum reflects anisotropic elongation or flattening. Define the skewness as:

$$OTS=\frac{\left(\lambda 3-\lambda 2\right)-\left(\lambda 2-\lambda \right)}{\lambda 3+\lambda 2+\lambda }$$

A high positive or negative *OTS* indicates directional shape asymmetry that can arise from twisted or irregular geometries—hallmarks of chiral macromolecular architecture.

Chirobiophore Vector Representation

Collectively, the descriptors defined above form the components of a single Chirobiophore vector:

C⃗  = [LTA, HPC, AES, DDP, LAI, OTS}

This vector captures a hierarchy of chiral features:

Local (atomic level): LTA, AES;

Mesoscale (secondary structure): HPC;

Membrane-scale (orientation and embedding): DDP, LAI;

Global (shape and symmetry): OTS.

By design, the Chirobiophore vector is frame-invariant and scale-aware, allowing for the quantitative comparison of chirality across proteins with diverse sizes, folds, and membrane orientations. It serves as the foundation for downstream tasks such as embedding analysis, classification, and structure–function correlation.

### 2.6. Membrane Comparison Descriptors

In addition to intrinsic chirality descriptors that apply broadly across biological macromolecules, membrane-associated proteins exhibit distinct geometric and topological asymmetries arising from their spatial embedding within lipid bilayers. Traditional stereochemical measures or secondary-structure-based indices do not fully capture these asymmetries. To address this, we define a suite of membrane comparison descriptors that quantify how chirality is expressed and explicitly modulated in the context of the membrane environment. These descriptors include the Depth Distribution Profile (DDP), the Leaflet Asymmetry Index (LAI), and the Orientation Twist Score (OTS). Each of these components captures different aspects of how structure and chirality are distributed relative to the bilayer’s spatial and functional axes.

#### 2.6.1. Depth Distribution Profile (DDP)

Conceptual Basis: The Depth Distribution Profile (DDP) captures how atoms or residues are distributed along the membrane’s normal vector—usually taken as the *Z*-axis in membrane-aligned coordinate systems. It quantifies asymmetry in insertion depth, which may reflect polarized function, selective exposure, or directional embedding [27].

Computational Method: Let ${\left\{Z_{i}\right\}}_{i=1}^{N}$ be the Z-coordinates of all relevant atoms or residue centroids (e.g., Cα atoms). We define a normalized histogram *P*(*z*) that represents the density of atoms across the bilayer:

$$P\left(z_{k}\right)=\frac{N_{k}}{N},$$

where *N_k_* = number of atoms in bin *k*.

The central tendency and spread of this distribution are characterized by the first three moments:

Mean

$$\mu z=\sum_{k}z_{k\cdot P\left(z_{k}\right)}$$

*μ_z_*: The expected value (mean) of the random variable *z*.

*z_k_*: A specific value that the variable *z* can take.

*P*(z_k_): The probability that *z* takes the value *z_k_*.

The sum is the overall possible value of *z_k_*.

*Skewness* (asymmetry of distribution):

$$Skewness=\frac{1}{\sigma_{z}^{3}}\sum_{k}{\left(z_{k}-\mu_{z}\right)}^{3}\cdot P\left(z_{k}\right)$$

where:

*μ_z_*: The mean (expected value) of the random variable *z*, computed as

$$\mu_{z}=\sum_{k}z_{k}\cdot P\left(z_{k}\right)$$

*σ_z_*: The standard deviation of *z*, computed as

$$\sigma_{z}=\sqrt{\sum_{k}{\left(z_{k}-\mu_{z}\right)}^{2}\cdot P\left(z_{k}\right)}$$

*z_k_*: Possible values of the random variable.

*P*(*z_k_*): The probability of value *z_k_*.

*Kurtosis* (peakedness):

$$Kurtosis=\frac{1}{\sigma_{z}^{4}}\sum_{k}{\left(z_{k}-\mu_{z}\right)}^{4}\cdot P\left(z_{k}\right)$$

where:

*z_k_* are discrete depth values;

*P*(*z_k_*) is the probability (or frequency) of *z_k_*;

*μ_z_* is the mean (expected depth);

*σ_z_* is the standard deviation of the depth values.

The DDP score can be taken as a vector or scalar combination of these statistics, providing a compact representation of depth-based asymmetry:

*DDP* = [*μz*, *Skewness*, *Kurtosis*]. This vector captures:

*μ_z_*: Central tendency (average depth);

*Skewness*: Asymmetry of depth values;

*Kurtosis*: Peakedness and tail heaviness.

To reduce this 3D descriptor to a scalar—useful for ranking, similarity, or thresholding the Euclidean norm of the *DDP* vector can be computed:

$$\left\|DDP\right\|\;=\sqrt{\mu_{z}^{2}}+{Skewness}^{2}+{Kurtosis}^{2}$$

This scalar serves as a compact score reflecting the overall statistical shape of the depth profile.

#### 2.6.2. Leaflet Asymmetry Index (LAI)

Conceptual Basis: Many membrane proteins exhibit leaflet-specific distributions of structural features, such as loops, charged residues, or cofactor sites. This asymmetry aligns with biological polarity (e.g., apical vs. basal surfaces) and underlies phenomena like signal propagation, ion gating, or vesicle fusion. The Leaflet Asymmetry Index (LAI) quantifies the imbalance in chiral center or motif density across the upper and lower leaflets of the membrane [28].

Definition: Partition the protein structure using the membrane midplane (typically z = 0 into:

Upper leaflet (extracellular): zi > 0 z_i > 0 zi > 0;

Lower leaflet (cytoplasmic): zi < 0 z_i < 0 zi < 0.

Let NupperN and Nlower be the number of identified chiral centers (or other motifs) in the upper and lower membrane regions, respectively (partitioned by the membrane midplane, typically at z = 0 z = 0 z = 0).

Then the *LAI* is defined as:

$$LAI=\frac{N_{upper}-N_{lower}}{N_{upper}+N_{lower}}$$

Interpretation:

LAI = 0 → symmetric distribution;

LAI > 0 → extracellular bias;

LAI < 0 → cytoplasmic bias.

Extensions: Weighted versions of LAI can account for chirality magnitude (e.g., LTA or AES values), using:

This is a weighted Leaf Asymmetry Index (LAI_weighted), based on chirality scores at different sites. The formula is:

$${LAI}_{weighted}=\frac{\sum_{i\in upper}\omega_{i}-\sum_{j\in lower}\omega_{j}}{\sum_{i\epsilon upper}\omega_{i}+\sum_{j\epsilon lower}\omega_{j}}$$

where:

$\omega_{i}$ is the chirality score at site I;

“upper” refers to sites where the chirality is considered upper-handed (e.g., right-handed, clockwise, or another designated category);

“lower” refers to sites with lower-handed chirality (e.g., left-handed, counterclockwise, or the opposite category).

Interpretation:

The numerator represents the net chirality bias, or the difference in the total score between the upper and lower groups.

The denominator represents the total chirality signal, which is the sum of both upper and lower scores.

The output will always be between −1 and 1, where:

1 means all signals are upper-handed;

−1 means all signals are left-handed;

0 means a perfect balance between upper and lower.

#### 2.6.3. Orientation Twist Score (OTS)

Conceptual Basis: Transmembrane helices often adopt cumulative torsional orientations, creating large-scale helical bundles or asymmetric packing arrangements. The Orientation Twist Score (OTS) measures the net angular rotation or torsional deformation across the bilayer, revealing membrane-spanning chirality trends. Orientation Twist Score (OTS), which quantifies the average directional tilt of membrane-spanning helices in a protein, weighted by their handedness [29].

Mathematical Definition: Let a unit vector represent each transmembrane helix v⃗ _i_ connecting its entry and exit points across the membrane (e.g., from the intracellular to the extracellular leaflet). Let θ_i_ be the twist angle of helix i, defined as the angle between the local helix axis and the global membrane normal z computed via:

θi = arccos(v⃗ i·z^)

v⃗ _i_: unit vector along the axis of the ith helix;

z^^^: unit vector in the membrane normal direction (usually taken as the *z*-axis).

So, θ_i_ is the angle between the helix and the membrane normal.

s_i_ = sign(torsion_i_)

This indicates whether the helix rotates right-handedly (+1) or left-handedly (−1), based on its torsion (e.g., derived from Cα backbone dihedrals).

Orientation Twist Score (OTS)

$$OTS=\frac{1}{n}\sum_{i=1}^{n}S_{i}\cdot \theta_{i}$$

n: number of membrane-spanning helices;

*s_i_·θ_i_*: combines direction (handedness) and tilt magnitude.

So OTS is the signed average tilt: right-handed tilts contribute positively, left-handed tilts negatively.

Furthermore: *s_i_* = sign(torsion_i_)

torsion_i_ refers to the backbone torsion of the *i*th membrane-spanning helix.

This torsion is typically computed from the dihedral angles along the Cα (alpha carbon) trace of the helix.

The sign of the torsion indicates the handedness of the helix:

*si* = +1: right-handed rotation;

*si* = −1: left-handed rotation.

Alternative Vectorial Formulation:

If we define twist vectors ⃗t_i_ in 3D space representing helical direction and rotation, a vectorial OTS can be calculated as:

$$O\underset{T}{\to }S=\frac{1}{n}\sum_{i=1}^{n}\underset{t_{i}}{\to }$$

The magnitude ||OTS⃗|| then reflects total twist intensity, while its direction encodes the net handedness axis.

Integration with the Chirobiophore

Together, the DDP, LAI, and OTS form a membrane-specific chirality subspace that complements the atomic- and fold-level descriptors (LTA, HPC, AES). These three descriptors provide essential insight into how chirality manifests across and within the bilayer, enabling a comparison of embedded systems in a biologically meaningful, geometry-aware manner [30].

The full Chirobiophore vector, extended to include these descriptors, is therefore:

C = [LTA, HPC, AES, DDP, LAI, OTS]

This 6-dimensional coordinate situates each membrane protein within a chirality space that captures both internal folding asymmetries and bilayer-related structural features.

The 6-dimensional Chirobiophore space provides a structured, composite framework that captures both molecular-level and membrane-level chirality features of a membrane protein. This space is defined by six key descriptors: LTA (Local Torsion Asymmetry), HPC (Helical Polarity Coherence), AES (Asymmetric Embedding Score), DDP (Directional Density Profile), LAI (Lipid Alignment Index), and OTS (Orientation Twist Score).

At the atomic scale, LTA quantifies asymmetry in local torsional angles across the protein backbone. A higher LTA value indicates more pronounced local geometric asymmetry, reflecting subtle chiral patterns in the fold. HPC operates at the fold level, capturing the alignment and handedness coherence of helical domains. Proteins with high HPC values exhibit consistent chiral architectures, such as uniformly right-handed α-helices. AES bridges the fold and membrane interface, measuring how asymmetrically a protein embeds into the lipid bilayer. High AES values suggest biased insertion or a tilted orientation relative to the membrane normal [31,32,33].

At the membrane level, DDP quantifies the distribution of protein mass along the membrane axis. It highlights structural tilt or density shifts within the bilayer. LAI focuses on the interface between membrane lipids and the protein, capturing the correlation between lipid tail orientation and protein structural features. A high LAI reflects a chiral alignment between lipids and the embedded protein. OTS, or Orientation Twist Score, is a vectorial measure that represents the average direction and intensity of twist across the bilayer. Its magnitude reflects the degree of twist, while its direction encodes the net handedness axis of the system [34].

Each protein can be represented as a unique point in this 6-dimensional space, with its coordinates [LTA, HPC, AES, DDP, LAI, OTS] [LTA, HPC, AES, DDP, LAI, OTS] [LTA, HPC, AES, DDP, LAI, OTS] forming a “chiral fingerprint.” The distance between points in this space reflects the similarity or divergence in chirality between proteins. Notably, the dimensions can be grouped conceptually: LTA, HPC, and AES capture fold-level chirality, while DDP, LAI, and OTS characterize membrane-level chirality. Together, these descriptors provide a comprehensive, geometry-aware representation of protein chirality in biologically relevant contexts [35].

### 2.7. Implementation

Each descriptor within the Chirobiophore framework is derived directly from the geometric structure of membrane proteins, as represented in their PDB (Protein Data Bank) coordinates. Notably, the calculation of these descriptors is performed independently of atom naming conventions, residue labels, or global coordinate frames. This design ensures that the framework remains robust across diverse protein structures and free from bias introduced by arbitrary orientation or labeling.

The Local Torsion Asymmetry (LTA) is computed using signed scalar triple products applied to backbone segments. This approach captures the handedness of local geometries by evaluating the chirality of atomic triplets, making it sensitive to subtle asymmetries in torsion angles along the protein chain.

For the Helical Polarity Coherence (HPC), the method involves computing the arctangent of vectors formed by normal vectors to planes defined by torsion angles. This descriptor quantifies the degree to which the helices maintain a consistent rotational sense, allowing for the detection of coherent chiral alignment across secondary structural elements.

The Asymmetric Embedding Score (AES) is derived from angular entropy. Specifically, it involves generating 2D or 3D angular histograms of protein atoms projected into bilayer-aligned coordinate systems. The entropy of these histograms then serves as a measure of how symmetrically or asymmetrically the protein is embedded relative to the membrane plane. A high entropy score corresponds to a more asymmetric orientation, revealing structural biases that may reflect functional asymmetry.

Membrane-level descriptors use spatially aware vector-based methods. The Directional Density Profile (DDP) is computed using projections of protein atom densities along the membrane normal, analyzed within sliding spatial windows that follow the bilayer axis. This approach detects longitudinal asymmetries, such as domain tilting or biased mass distributions.

The Lipid Alignment Index (LAI) measures the correlation between vectors representing lipid tail orientations and local protein geometry. By projecting both lipid and protein vectors into a membrane-centered frame, LAI captures how well lipid molecules align with chiral protein features—an indicator of structural compatibility and possible chiral induction at the interface.

Finally, the Orientation Twist Score (OTS) is determined by aggregating local twist vectors (each representing a helical direction and rotation) across the protein’s transmembrane domains. These vectors are averaged to produce a single vectorial quantity, where the magnitude reflects the overall twist intensity and the direction encodes the net handedness axis of the embedded protein.

Together, these computation methods enable automated, geometry-aware chirality analysis of membrane proteins that is scalable, label-agnostic, and sensitive to both local and global asymmetries. The resulting Chirobiophore vectors provide a powerful tool for comparative analysis, classification, and interpretation of membrane protein architecture in biologically meaningful terms [29,36,37,38].

### 2.8. Chirobiophore as a Fiber Bundel

We can interpret the Chirobiophore as a fiber bundle; the base space is the pro-tein/membrane’s primary structure. The fiber at each point encodes the local chirality state: Fx = {LTAx, HPCx, AESx, …} where LTAx = Local Tetrahedral Asymmetry at position x, HPCx Helical Path Curvature at position x, AESx = Asymmetric Environment Score at po-sition x, … = Potential additional descriptors (e.g., DDPx, LAIx, OTSx). This defines Fx as a local chirality vector—the fiber over base point xxx—in a fiber bundle interpretation, where the full protein or membrane structure becomes a field of such vectors across space. The whole structure is a field of chirality projected onto the protein. This allows us to treat chirality as a distributed field, not just a global label, similar to a vector field over a mem-brane topology [39].

### 2.9. Chirobiophore Graph Theory Representation

A protein/membrane can be viewed as a graph: Nodes: Residues or atoms, Edges: Bonds or interactions, Node weights: Local chirality values (LTA, AES, etc.). Then: Global chirality = integrated topological signature over the graph. Subgraph isomorphisms = conserved chiral motifs. Graph spectral analysis = identify dominant chirality modes. This enables topological persistence analysis (via persistent homology) to detect multiscale asymmetry [9,11].

### 2.10. Chirobiophore Vector

The Chirobiophore vector represents the core output of this framework, encapsulating the multidimensional nature of chirality in membrane proteins in a concise and interpretable format. This six-dimensional vector serves as a geometric and functional fingerprint, allowing for meaningful comparison, classification, and analysis of membrane protein structures based on chirality-specific features.

Formally, the vector is defined as:

C = [LTA, HPC, AES, DDP, LAI, OTS]

Each element of this vector corresponds to one of the six descriptors introduced previously, selected to span both atomic-level and membrane-level structural asymmetries. The components are computed from intrinsic geometry and are independent of atom names, residue labels, or protein orientation, making the vector robust and generalizable across diverse datasets.

#### 2.10.1. Fold-Level Components

Local Torsion Asymmetry (LTA): Quantifies fine-grained chirality at the atomic level by measuring asymmetry in torsion angles along the protein backbone. This is particularly sensitive to local structural irregularities and provides insight into intrinsic folding asymmetries, regardless of the membrane context.

Helical Polarity Coherence (HPC): Measures the consistency and alignment of helical elements with respect to their rotational polarity. A high HPC indicates that the helices share a common handedness and orientation, reflecting global fold regularity and potential functional coherence.

Asymmetric Embedding Score (AES): Captures the orientation and placement of the protein within the lipid bilayer by measuring entropy from angular histograms in membrane-aligned coordinates. A higher AES signifies a greater deviation from symmetric embedding, which can reflect biological phenomena such as unidirectional signaling or asymmetric domain exposure [31].

#### 2.10.2. Membrane-Level Components

Directional Density Profile (DDP): Reflects how protein mass is distributed along the membrane normal. By computing localized density projections, DDP identifies biases such as structural tilt, lopsided transmembrane domain arrangement, or mass asymmetry between extracellular and cytoplasmic regions.

Lipid Alignment Index (LAI): Evaluates how well the protein’s geometry aligns with the orientation of surrounding lipid tails. This captures protein–lipid interface chirality and may highlight structural adaptations for lipid recognition, stabilization, or modulation.

Orientation Twist Score (OTS): Provides a global measure of twist across the bilayer. It is computed as the vector average of local helical twist directions. The magnitude of OTS reflects the degree of net helical deformation, while its direction encodes the axis of net handedness, offering a holistic view of membrane-twist architecture.

By placing a membrane protein in this six-dimensional space, the Chirobiophore vector characterizes its complete chirality profile, bridging molecular folding properties and membrane interactions. The vector can be used as a coordinate system for comparing proteins: structural similarity in chirality corresponds to proximity in this space. Furthermore, because the Chirobiophore vector supports metric operations, standard techniques such as clustering, principal component analysis, and similarity scoring can be applied.

This enables not just pairwise comparison, but also systematic analysis across protein families, functional groups, or evolutionary lineages, with chirality treated as a first-class structural descriptor. The Chirobiophore space can also be integrated into machine learning workflows, facilitating classification tasks, anomaly detection, and interpretability in structural prediction pipelines.

Notably, the inclusion of both fold-level and membrane-level descriptors ensures that the vector captures the multiscale nature of chirality in membrane proteins—something traditional shape-based or sequence-based methods often overlook. Thus, the Chirobiophore vector stands as a biologically informed, geometrically rigorous tool for advancing our understanding of protein structure, function, and interactions in membrane environments [40,41,42].

### 2.11. Chirobiophore as a Point in Chirality Space

C = [LTA, HPC, AES, DDP, LAI, OTS] ∈ ℝ^6^

This forms a 6D coordinate, where each dimension represents a distinct type of chirality or asymmetry. Every structure (protein, membrane, model) is a point in this chirality space.

Each Chirobiophore vector defines a position in a six-dimensional real-valued vector space:

C = [LTA, HPC, AES, DDP, LAI, OTS] ∈ R^6^

This mathematical formulation situates every protein, membrane-embedded system, or computational model as a single point in a continuous chirality space, where each axis reflects a distinct and biologically interpretable form of asymmetry. In essence, the Chirobiophore vector acts as a coordinate system for quantifying and comparing structural chirality at multiple spatial scales.

Each of the six dimensions—LTA, HPC, AES, DDP, LAI, and OTS—serves as an orthogonal descriptor, contributing independent information about the structure’s handedness. The first three dimensions (LTA, HPC, AES) capture intrinsic asymmetries of the protein’s fold and secondary structure, while the latter three (DDP, LAI, OTS) encode how the protein interacts with and is organized within the lipid bilayer. This split ensures that the space reflects both internal chiral architecture and extrinsic membrane-contextual asymmetry [43,44].

By embedding a protein in this chirality space, its position vector becomes a comprehensive structural signature. Proteins with similar chiral properties will cluster nearby in this space, whereas those with distinct folding or embedding asymmetries will occupy more distant locations. This geometric representation supports both qualitative reasoning (e.g., visualizing chirality shifts) and quantitative analysis (e.g., computing Euclidean or cosine distances between vectors).

Importantly, this abstraction enables a wide range of computational applications:

Clustering and classification: Proteins can be grouped based on proximity in chirality space, facilitating the discovery of structural or functional families that share chiral motifs.

Outlier detection: Unusual structures—such as those resulting from mutations, design errors, or alternate topologies—may occupy sparsely populated or extreme regions of the space.

Time-dependent studies: In simulations, the evolution of a protein’s Chirobiophore vector over time can reveal conformational drift, folding pathways, or membrane reorganization.

Comparative modeling: The Chirobiophore space provides an objective basis for comparing experimental structures to computational models or predictions, offering chirality-based validation metrics [45,46].

Moreover, because each axis of the space is derived from raw geometry, independent of labeling, sequence, or alignment, the representation is broadly applicable across organisms, resolutions, and modeling methods. The vectorization process is also invertible in the sense that a location in chirality space can be mapped back to interpretable physical features (e.g., “this protein has high twist but low polarity coherence”).

In this way, the Chirobiophore space serves not merely as a descriptive tool but as a conceptual framework for exploring the geometry of membrane protein chirality. It bridges the gap between molecular structure and higher-level organization, allowing chirality to be treated as a first-class structural property—measurable, comparable, and computationally actionable.

In addition to its metric and geometric properties, the Chirobiophore space can be understood from a topological perspective, offering a more abstract but powerful layer of interpretation. By treating chirality as a property that is invariant under continuous deformation, topology enables us to reason about structural class, global orientation, and symmetry breaking in ways that transcend local atomic fluctuations or alignment conventions [47].

At its core, the Chirobiophore vector defines a coordinate embedding of protein structures into a real-valued topological vector space, where open sets correspond to regions of similar chirality, and neighborhood structure reflects the continuity of geometric transformation. That is, small perturbations in fold or membrane placement result in continuous changes in the Chirobiophore vector. This continuity ensures the stability of the representation under biologically plausible structural variations—such as domain flexing, side-chain dynamics, or lipid bilayer deformation—preserving topological class membership even when exact geometry changes.

From a topological classification standpoint, proteins that occupy the same connected region (or component) of Chirobiophore space can be interpreted as chirality-homologous—that is, they belong to the same structural chirality class, even if their atomic details differ. This is particularly useful for understanding evolutionary variation, conformational ensembles, or functional switching (e.g., gating in ion channels), where chirality might shift locally but the topological identity remains intact [48].

Moreover, several Chirobiophore components themselves are inherently topological in nature:

HPC reflects winding number consistency, a concept that parallels topological invariants like twist or linking number in knot theory. Although termed “Helical Path Curvature,” the HPC descriptor should be interpreted as a measure of torsional handedness and coherence along the backbone. The terminology reflects the cumulative geometric behavior of the path, but does not correspond to curvature in the formal differential geometric sense.

OTS encodes net helical handedness direction, acting similarly to an orientation vector in a fiber bundle or a mapping degree in topology.

AES and DDP, though computed geometrically, are sensitive to global asymmetry and can identify transitions across topological barriers—such as inversion or domain flipping—within the bilayer.

These properties make Chirobiophore space especially suited for capturing topological transitions, such as symmetry-breaking events, pseudo-mirror states, or chiral inversion. For example, if a protein undergoes a change in embedding that mirrors its transmembrane domains (as might occur during dimerization or misfolding), this would correspond to movement across a chirality-inverting manifold in the space—a topologically significant shift, even if the total structure remains energetically favorable.

Importantly, because the Chirobiophore space is homeomorphic to R^6^, it admits continuous mappings, embeddings, and vector field definitions, allowing techniques from topology and differential geometry to be applied. For instance, chirality gradients (i.e., derivatives of Chirobiophore values across spatial or temporal dimensions) can define vector flows or morphogen-like landscapes for structural organization [49,50].

Implications

Understanding the Chirobiophore vector as a topological embedding reinforces its role not just as a structural descriptor but as a model of continuity, class identity, and deformation resilience in membrane protein structure. It allows one to move beyond static comparison toward a dynamical, deformation-aware view of chirality—ideal for modeling folding pathways, membrane transitions, or evolutionary divergence.

This perspective also opens the door to topology-based learning and analysis techniques, such as persistent homology, topological clustering, and manifold learning, which can reveal higher-order structure in the distribution of proteins across Chirobiophore space—discovering not just groups, but also shape in the chirality space itself [51].

### 2.12. Chirality Manifold: A Curved Shape Space

**Figure 2:**
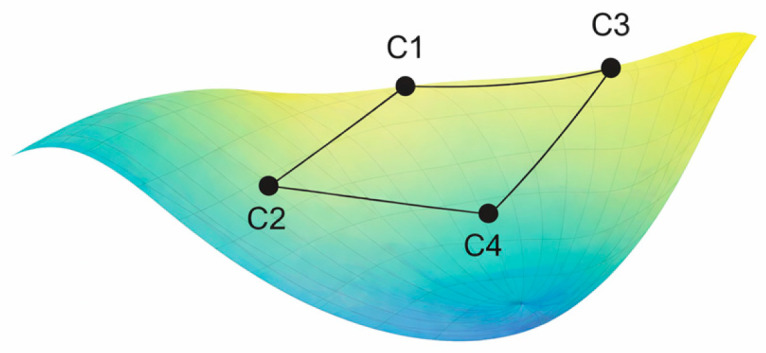
Chirality manifold. C_1_ → C_2_ → C_3_ → C_4_ could represent a developmental transition (e.g., from primitive to specialized endothelial cells).

Imagine all physically possible structures as forming a curved, nonlinear manifold inside ℝ^6^ (Figure 2)

The curvature shows that not all chirality states are equally likely or accessible.

Although the Chirobiophore vector maps each membrane protein to a point in a six-dimensional Euclidean space (R^6^), the set of physically realizable protein structures does not fill this space uniformly. Instead, these biologically plausible configurations occupy a curved, lower-dimensional manifold embedded within the larger coordinate space. This conceptual space, referred to as the chirality manifold, represents a continuous, non-linear surface through which proteins can transition via structural deformation, functional adaptation, or evolutionary change.

The chirality manifold reflects the constraints imposed by the biophysics of protein folding, membrane embedding, and functional specialization. Because protein structures are governed by steric limits, thermodynamic feasibility, and membrane interactions, only a subset of all mathematically possible Chirobiophore vectors corresponds to viable molecular configurations. In this view, each protein exists not as an isolated point in R^6^, but as a location on a smooth, possibly curved hypersurface that defines the space of allowable chiral structures [52,53].

This manifold offers a robust conceptual framework for understanding how chirality evolves and is regulated across various structural, developmental, and evolutionary trajectories. For example, a sequence of states represented by vectors C1 → C2 → C3 → C4 might correspond to a biological transformation, such as the differentiation of membrane-bound receptors in primitive cells into highly specialized forms in vascular endothelium. The pathway across the manifold captures not just discrete chirality values but also their smooth transitions, revealing which structural changes are energetically or evolutionarily accessible.

Importantly, the curvature of the manifold is biologically informative. In regions of low curvature—where the surface is relatively flat—small changes in protein structure lead to proportionally small changes in chirality. These areas represent standard, stable, or low-energy configurations that many proteins may share. In contrast, regions of high curvature indicate rare, transitional, or energetically unfavorable states, where minor geometric perturbations produce significant shifts in chirality. Such regions may correspond to intermediate states during folding, moments of functional switching, or structures prone to instability or misfolding [54].

This topological view also explains why specific chirality profiles are more prevalent or evolutionarily favored than others: proteins tend to occupy broad, accessible regions of the manifold, while highly twisted, asymmetrically embedded, or otherwise extreme configurations are rare and often short-lived. Thus, chirality becomes not just a descriptor, but a constrained evolutionary variable with its own geometric logic.

In parallel to the continuous manifold, a graph-theoretical representation of chirality can be used to capture local structure and connectivity. In this representation, each protein is modeled as a graph, where nodes represent residues or atoms, and edges denote covalent bonds or spatial proximity within a defined cutoff. These edges can be weighted using chirality-relevant metrics—such as Local Torsion Asymmetry (LTA), Asymmetric Embedding Score (AES), or a Side-chain Orientation Coherence (SOC) metric—thus allowing chirality to be encoded into the topology of the graph itself [55].

Analyzing such graphs enables the application of powerful topological data analysis methods. Clustering algorithms can identify recurring chiral motifs, such as twisted β-sheets or helical bundles with consistent handedness. Betti numbers, a concept from algebraic topology, can be computed to count the number of loop-like structures or cavities in the graph, which correspond to structural patterns such as twists, folds, and tunnels. Additionally, persistent homology—a method that tracks how topological features persist across multiple spatial or energetic scales—can reveal stable chiral cores in the protein, distinguishing them from transient or noise-prone conformations.

When combined, the chirality manifold and the graph representation offer a multiscale, dual-view framework for understanding membrane protein chirality. The manifold captures the global, continuous variation of chirality across the protein space, enabling comparative analysis and trajectory modeling. The graph captures local, discrete structure, suitable for motif detection, topological invariants, and structural classification.

Together, these views provide a rich geometric and topological foundation for investigating the role of chirality in protein function, stability, and evolution. They open new avenues for structural prediction, synthetic design, and classification based not only on shape or sequence, but on the profound structural asymmetries that govern how proteins fold, embed, and operate in the membrane environment [56,57,58].

### 2.13. Chirality Fiber Bundle (Field over Structure)

To move beyond a global, vector-based representation of chirality, we can introduce a fiber bundle model, which conceptualizes chirality not as a single value for an entire protein, but as a distributed field mapped across its structure. In this view, the protein is treated as a base space—a continuous curve or surface defined by its atomic or residue-level geometry—and each point along this base is associated with a local chirality vector, forming a fiber attached to that point.

For example, consider a membrane protein backbone. At each residue iii, one can compute a local chirality descriptor, such as:

Residue i → [LTA_i_, AES_i_]

Here, Local Torsion Asymmetry (LTA) and Asymmetric Embedding Score (AES) provide local measures of handedness and membrane asymmetry, respectively. These per-residue vectors form a field of chirality descriptors aligned along the backbone. Visually, this can be imagined as a series of small arrows (vectors) “attached” to each point of the structure, pointing in the direction and magnitude of local chirality:

This construction defines a chirality fiber bundle, in which each “fiber” (the local vector) varies smoothly or discontinuously along the base structure (the protein). The resulting field can be analyzed similarly to a topographic map, revealing patterns of chiral intensity, inversion, or coherence along the structure.

**Figure 3:**
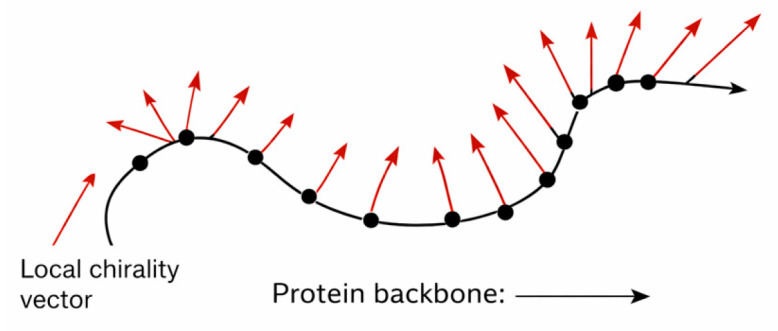
Chirality fiber bundle representation along a protein backbone. The protein backbone (black curve, arrow indicating sequence direction) represents the base space of the bundle. At each residue position, a local chirality vector (red arrows) encodes per-residue chirality descriptors (e.g., Local Torsion Asymmetry, LTA, and Asymmetric Embedding Score, AES). The collection of these smoothly varying local vectors defines a distributed chirality field over the structure, enabling analysis of regional asymmetry, structural transitions, and functional domain boundaries.

This localized view is handy for studying heterogeneous chirality within proteins—cases where some regions exhibit strong chirality and others are nearly symmetric. It also enables gradient-based analysis, where abrupt changes in vector orientation may signal structural boundaries, hinge points, or transitions between functional domains (Figure 3) [9,59].

#### Loops and Bifurcations in Chirobiophore Trajectories

When the Chirobiophore vector is tracked over time, along a conformational pathway, or across evolutionary states, it traces a trajectory through chirality space. These trajectories can exhibit rich geometric and topological structure, including loops, branches, and bifurcations.

**Figure 4:**
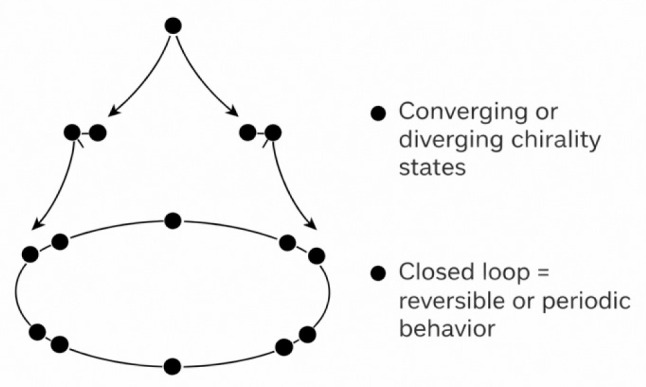
Loops, bifurcations, and convergence in Chirobiophore trajectories. Each black node represents a distinct chirality state in Chirobiophore space. Directed edges indicate transitions along a conformational, developmental, or evolutionary pathway. The upper branching structure illustrates bifurcation and divergence, where a single chirality state evolves into multiple alternative configurations. Converging nodes represent structural convergence toward a shared chirality profile. The lower closed loop depicts reversible or periodic dynamics, corresponding to cyclical structural transitions such as allosteric switching or recurring functional states. These topological patterns encode biologically meaningful dynamics within the chirality manifold.

A loop in Chirobiophore space occurs when a protein returns to a previous chirality state after undergoing a structural change. This might correspond to reversible transitions, such as those seen in allosteric regulation, conformational switching (e.g., active vs. inactive states of a channel), or cyclical tissue processes like inflammation, repair, and homeostasis. The loop structure in chirality space reflects periodicity or recurrence in the underlying physical system (Figure 4).

A bifurcation represents a point at which a single trajectory splits into two or more paths, reflecting a decision point in chirality. This can arise in evolutionary divergence, structural polymorphism, or during protein folding when intermediate states may resolve into distinct final forms. Conversely, convergence occurs when multiple distinct structures evolve toward a common chirality state—perhaps due to functional constraints or shared membrane embedding conditions.

These loop and branching behaviors are not merely mathematical curiosities—they capture biologically meaningful dynamics. For instance, A loop may encode the reversible morphodynamics of a signaling protein toggling between resting and active states. A bifurcation might model tissue-specific adaptation, where the same protein adopts different chiral conformations in different cellular environments. Converging branches could reflect structural convergence, where unrelated proteins adopt similar chiral profiles to interface with a common membrane lipid domain. By analyzing these trajectories within Chirobiophore space, researchers can uncover hidden structure–function relationships, identify points of structural flexibility or constraint, and map out dynamic chirality landscapes that are informative for both biology and design (Table 1). The scripts used to compute and demonstrate the Chirobiophore are attached to Supplementary Materials S1 [60,61,62,63].

Future validation efforts will focus on applying the Chirobiophore framework to expanded membrane protein datasets, including homologous families, conformational ensembles derived from molecular dynamics simulations, and experimentally annotated membrane orientation databases. Such studies will enable statistical characterization of descriptor distributions and more rigorous assessment of robustness and generality. Comprehensive external validation, including benchmarking across larger and more diverse datasets and comparison with established chirality measures, is an important direction for future work and lies beyond the scope of the present study.

## 3. Results

We applied the chirobiophore approach to human endothelial and Artemia salina membrane protein models. Despite having a similar topology, the systems exhibited distinct HPC profiles, suggesting species-specific structural asymmetry. AES revealed localized packing complexity not evident in global models. Both systems had minimal net LTA, consistent with symmetric atomic geometry. DDP and LAI revealed non-uniform embedding and leaflet bias, while OTS detected distinct transmembrane twist behaviors [9]. All Chirobiophore descriptors are dimensionless quantities derived from normalized geometric measures unless otherwise explicitly stated. The level of detail presented in this section is intentional, intended to provide a transparent and reproducible characterization of descriptor behavior across structurally diverse systems. These results provide an initial internal validation by demonstrating that the descriptor set consistently distinguishes structurally and functionally distinct protein classes within the defined scope.

### 3.1. The Chirobiophore Manifold

Each structure’s Chirobiophore vector C ∈ R^6^ exists in a non-Euclidean, nonlinear subspace of chirality metrics: the whole space of possible vectors forms a chirality manifold M ⊂ R^6^. This space has local curvature—some combinations of metrics are biologically impossible or unstable. Topological clustering reveals basins of biologically realizable chirality states. This allows mapping trajectories, e.g., structural evolution from primitive to more biologically more evolved and specialized membranes, as paths on the manifold [64].

### 3.2. Topological Invariants and Chirality

We can compute topological invariants that define the “shape” of chirality: Betti numbers: Count the number of n-dimensional holes in chirality fields (e.g., closed loops of twist). Euler characteristic: Describes the net structure of the chirality domain boundaries. Genus (g): The number of holes/tunnels in a 3D chirality distribution. These abstract metrics link geometry to topology and can classify chirality into homotopy classes (i.e., topologically similar vs. fundamentally different) [65].

### 3.3. Example: Chirality Pathways as Topological Loops

A developmental process (e.g., angiogenesis) can be represented as a trajectory through a Chirobiophore. The path may loop or spiral, forming a topological cycle. Closed loops = periodic structures (e.g., cyclic remodeling). Branching = bifurcations in developmental fates [66].

#### 3.3.1. Topological Extension of the Chirobiophore

##### Chirality Persistence Score (CPS)

Inspired by: Persistent homology. Definition: Measures how long chiral features (loops, cavities, asymmetry patterns) persist across spatial scales during coarse-graining. Method: Build a residue/atom graph. Encode local chirality (e.g., LTA or AES) as node weights. Apply persistence analysis to detect “birth” and “death” of topological features (e.g., twist loops).

$CPS=\sum_{i=1}^{N}(d_{i}-b_{i})$, High CPS → stable, robust chiral architecture. Low CPS → transient, disordered chirality [52].

##### Chirality Genus Index (CGI)

Inspired by: Surface topology. Definition: Measures the number of “holes” or “handles” in the spatial distribution of chirality fields.

$CGI=1-\frac{1}{2}\left(V-E-F\right)$, where V, E, and F are vertices, edges, and faces in a surface mesh of chirality intensity. Interpretation: High genus = toroidal or multi-domain chirality (complex), Low genus = planar or unidirectional chirality (simple) [67].

##### Chirality Vector Field Curl (CVF)

Inspired by: Differential geometry. Definition: Captures the rotational tendency of chirality fields (e.g., membrane twist or helical winding). Let each residue/patch have a chirality vector (e.g., direction of asymmetry), then CVF = ×C⃗. ∇ × C⃗  Interpretation: High CVF = swirling, twisted domains (e.g., transporters, channels) Low CVF = linear chirality flow (e.g., structural membranes) [68].

##### Chirality Entanglement Index (CEI)

Inspired by: Knot theory. Definition: Measures how intertwined different chiral pathways or helices are in space. $CEI=\frac{1}{N}\sum_{I<J}Lk\left(i,j\right)$, where Lk(i,j) is the Gauss linking number between chirality paths (e.g., α-helices or domain axes). Interpretation: High CEI = deeply interwoven chirality (e.g., GPCRs, ion channels). Low CEI = separable domains [69].

##### Topological Chirality Complexity (TCC)

Inspired by: Fractal dimension & topological entropy. Definition: Captures the overall topological entropy of chirality fields, based on the unpredictability of transitions between local chiral states. $TCC=-\sum_{i=1}^{N}p_{i}log\left(p_{i}\right)$, where *p_i_* are the transition probabilities between local chirality states across the structure. Interpretation: High TCC → information-rich chirality topology (signal processing, gating) Low TCC → redundant or symmetric chirality fields [70].

The table below summarizes each descriptor (Table 2).

### 3.4. Validation Results

The summarizing table of the computed chirobiophore vectors and their values is listed in the table below (Table 3):

Here are the full Chirobiophore descriptor values for 1C3W (bacteriorhodopsin) (Table 4):

Here are the full Chirobiophore descriptor values for 1F88 (Rhodopsin) (Table 5).

Here are the full Chirobiophore descriptor values for 1J4N (Aquaporin-1) (Table 6).

**Figure 5:**
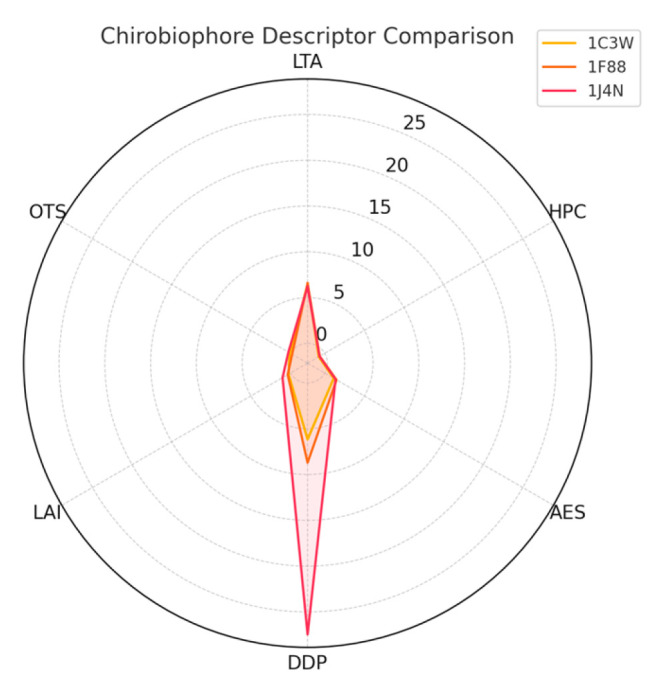
The Chirobiophore radar plot comparing the three canonical α-helical membrane proteins (1C3W, 1F88, and 1J4N). Each axis represents one of the six chirality descriptors.

In Figure 5, the Chirobiophore descriptor comparison is shown.

- 1J4N stands out with a significantly higher DDP and LAI, indicating profound asymmetry and strong leaflet bias. 1F88 exhibits elevated AES and moderate negative OTS, indicating local packing irregularity and a left-handed twist.
- 1C3W has relatively balanced chirality metrics with a mildly right-leaning OTS. Radar plot of Chirobiophore descriptors for [protein group]. All six descriptors (LTA, HPC, AES, DDP, LAI, OTS) are shown after z-score standardization (mean = 0, standard deviation = 1) across the analyzed dataset, allowing direct visual comparison of relative descriptor magnitudes. All axes are dimensionless and comparable by construction.

Here are the full Chirobiophore descriptor values for 2RH1 (β2-Adrenergic Receptor) (Table 7).

Here are the full Chirobiophore descriptor values for 3ODU (CXCR4 Chemokine Receptor) (Table 8).

Here are the full Chirobiophore descriptor values 4DKL (Table 9).

**Figure 6:**
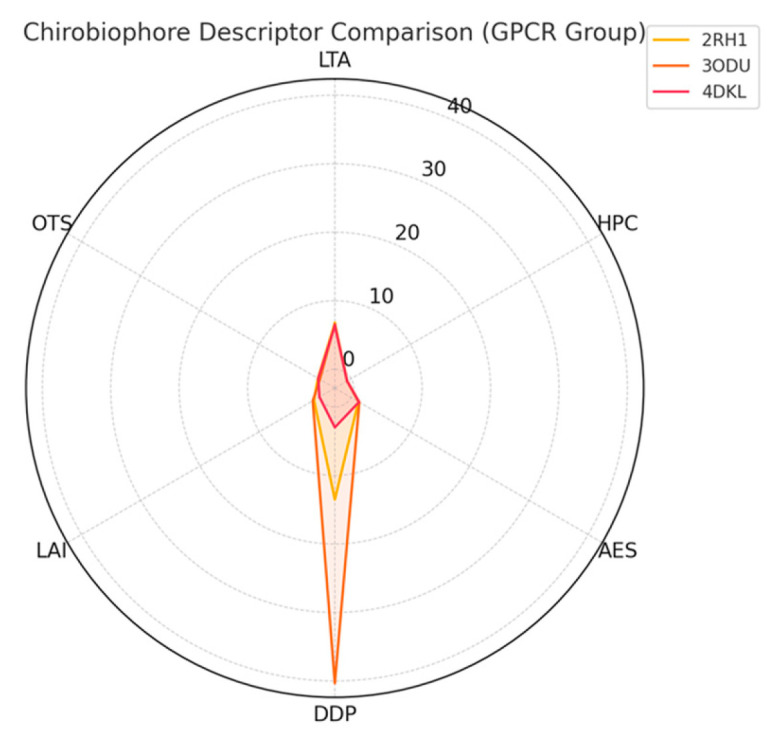
The radar plot for the GPCR group (2RH1, 3ODU, 4DKL) shows how each protein expresses chirality across the six Chirobiophore descriptors. 3ODU stands out with extremely high DDP and LAI. 2RH1 has a balanced but elevated chirality profile. 4DKL exhibits the lowest DDP and a negative LAI, indicating a slightly lower-leaflet bias. Radar plot of Chirobiophore descriptors for [protein group]. All six descriptors (LTA, HPC, AES, DDP, LAI, OTS) are shown after z-score standardization (mean = 0, standard deviation = 1) across the analyzed dataset, allowing direct visual comparison of relative descriptor magnitudes. All axes are dimensionless and comparable by construction.

Figure 6 shows the Chirobiophore descriptor comparison—GPCR group.

Here are the full Chirobiophore descriptor values for 2A79 (Voltage-Gated Potassium Channel) (Table 10).

Here are the full Chirobiophore descriptor values for 1C17 (ATP Synthase Subunit c) (Table 11).

Here are the full Chirobiophore descriptor values for 1OTS (ClC Chloride Transporter) (Table 12).

**Figure 7:**
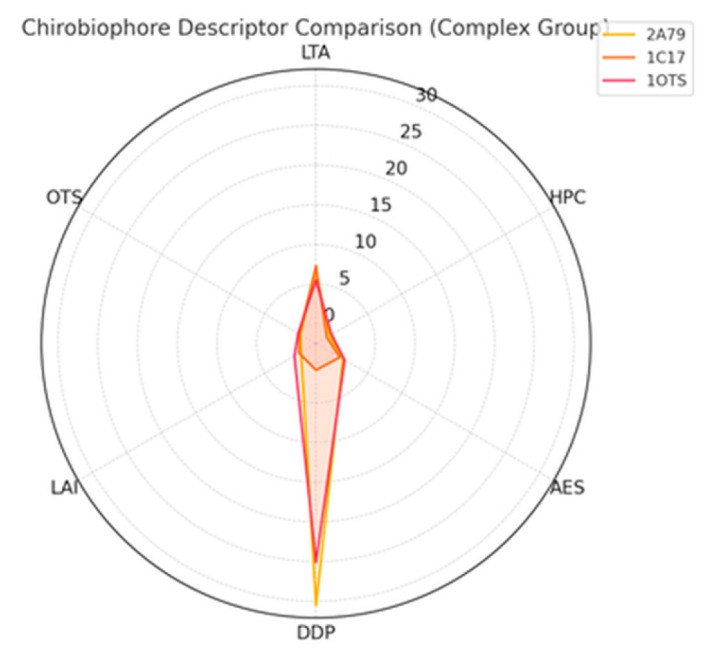
The radar plot for the complex membrane protein group (2A79, 1C17, 1OTS).

Figure 7 shows the Chirobiophore descriptor comparison complex membrane proteins.

- 1C17 shows the highest LTA and HPC, indicating strong local and global chirality, but is symmetric in depth and leaflet embedding. 2A79 exhibits strong DDP and AES, with bias toward the lower membrane leaflet. 1OTS has the highest AES of any protein analyzed so far, signaling extreme local packing irregularity. Radar plot of Chirobiophore descriptors for [protein group]. All six descriptors (LTA, HPC, AES, DDP, LAI, OTS) are shown after z-score standardization (mean = 0, standard deviation = 1) across the analyzed dataset, allowing direct visual comparison of relative descriptor magnitudes. All axes are dimensionless and comparable by construction.

**Figure 8:**
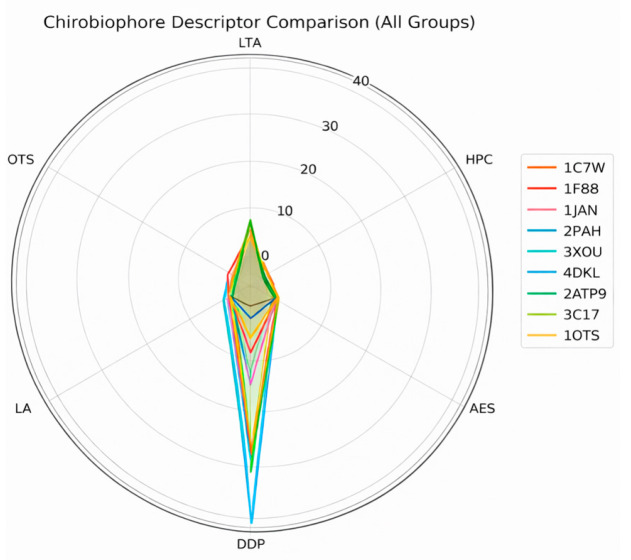
The radar plot compares all three groups of membrane proteins across the six Chirobiophore descriptors: Canonical proteins (1C3W, 1F88, 1J4N) show relatively balanced chirality profiles with modest embedding asymmetries. GPCRs (2RH1, 3ODU, 4DKL) are characterized by higher DDP and LAI, reflecting their asymmetric interaction with the membrane. Structurally complex proteins (2A79, 1C17, 1OTS) exhibit the most diverse profiles. Notably, 1C17 leads in LTA and HPC, but is nearly symmetric in embedding, while 1OTS has the highest AES, indicating local geometric disorder. Radar plot of Chirobiophore descriptors for [protein group]. All six descriptors (LTA, HPC, AES, DDP, LAI, OTS) are shown after z-score standardization (mean = 0, standard deviation = 1) across the analyzed dataset, allowing direct visual comparison of relative descriptor magnitudes. All axes are dimensionless and comparable by construction.

All proteins form three groups and are represented together in the figure below (Figure 8).

## 4. Discussion

Chirobiophore establishes a new paradigm for describing and comparing structural chirality in biomolecules.

This new paradigm emerged from the intersection of three significant contributions in the SAR/QSAR research domain:

(a) The concept of pharmacophore developed by Lemont B. Kier in the 1960s. Kier’s pioneering work on the molecular orbital calculation of preferred conformations of acetylcholine, muscarine, and muscarone laid the foundation for the pharmacophore concept, which is central to understanding molecular interactions in drug design (Kier, 1967; Kier, 1971) [71,72,73].
(b) The development of biodescriptors by Basak et al. which maps biologically complex objects such as DNA sequences and proteomics maps to sets of real numbers and/or vectors. Key contributions from Basak include the role of mathematical chemodescriptors and proteomics-based biodescriptors in drug discovery (Basak, 2010), as well as the mathematical descriptors of DNA sequences (Nandy et al., 2006), proteomic maps (Vracko & Basak, 2004), and the study of chemically induced changes in proteomes (Randic et al., 2001) [74,75,76,77].
(c) The formulation of multidimensional spaces of numerical chirality descriptors using graph theory, beginning with the CIP (Cohn-Ingold-Prelog) rules of structural stereochemistry. Notable contributions in this area include the work by Natarajan, Basak, and Neumann (2007), as well as recent developments in chirality descriptors for structure–activity relationship modeling of bioactive molecules (Natarajan et al., 2024) [78,79].

It is generalizable, extensible, and bridges the gap between atomistic detail and functional insight. Future work will expand the descriptor set (e.g., 3D entropy, Zernike moments) and apply it to dynamic simulations. The Chirobiophore can be used for

(a) Angiogenesis tracking, chirobiophore vectors change across endothelial developmental phases;
(b) Biologics comparison: structural biosimilarity can be assessed via chirality vectors;
(c) Drug design: membrane protein binding pockets can be classified by local chiral asymmetry; membrane asymmetry studies: DDP and Lai enable tracking leaflet-specific insertion and orientation patterns [80].

A central conceptual clarification of the present framework concerns the distinction between geometric asymmetry and chirality. While asymmetry broadly denotes any deviation from symmetry, chirality is a stricter geometric property defined by non-superimposability under mirror reflection, and therefore not all asymmetric structures are chiral. In the Chirobiophore framework, chirality is not attributed to any single descriptor in isolation, but is instead treated as an emergent, multiscale property arising from the combined configuration of complementary geometric features. Descriptors such as Local Tetrahedral Asymmetry (LTA) and Helical Path Curvature (HPC) directly encode handedness at atomic and backbone levels, whereas descriptors such as Directional Density Profile (DDP) and Leaflet Asymmetry Index (LAI) quantify directional or environmental asymmetries relative to the membrane context. These latter descriptors do not represent chirality independently, but contribute to the overall chiral organization of the system when interpreted jointly with torsional and geometric measures. This distinction is particularly relevant for membrane proteins, where embedding, orientation, and spatial anisotropy play a critical role in shaping functional structural asymmetry. By adopting a multivariate representation, the Chirobiophore framework captures distributed and emergent chirality across scales without reducing it to a single scalar quantity.

### 4.1. Chirobiophore Similarity & Clustering

Chirobiophoric similarity can be compared using vector norms: D(C1, C2) = ∑i(C1i − C2i)2D. PCA can be used to reduce dimensionality and visualize chirality clusters.

Clustering methods (k-means, DBSCAN) can be used to define families with similar chiral behavior.

ML models may be applied to predict function from structure via chirality signatures.

#### Potential for Expansion

The Chirobiophore space can be expanded with future descriptors like Zernike 3D moments—volumetric chirality; spherical harmonics—global shape asymmetry; membrane fluctuation asymmetry; and time-resolved twist metrics from MD simulations.

By framing the Chirobiophore topologically, one can compare global vs. local chirality, understand evolutionary constraints on structure, build morphological networks linking protein families, and explore chirality-driven function spaces mathematically rigorously [81] (Table 13).

**Figure 9:**
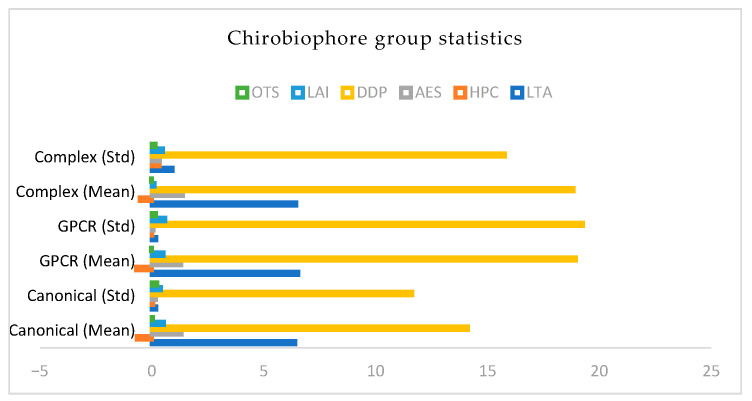
Chirobiophore group statistics.

Furthermore, characterizing the chemiobiophore as a group, statistics, the following graph represents the chemiobiophore descriptors (Figure 9).

LTA (chirality) is highest in the Complex group—mainly due to 1C17.

HPC (torsional coherence) is most negative (left-handed) in the Complex group as well, suggesting a pronounced helical regularity or bias.

AES (local disorder) peaks in the GPCR and Complex groups, consistent with asymmetric packing.

DDP and LAI are highest in the GPCR group, confirming strong membrane polarity.

OTS (twist) is mixed: GPCRs and Complex proteins show both positive and negative biases.

**Figure 10:**
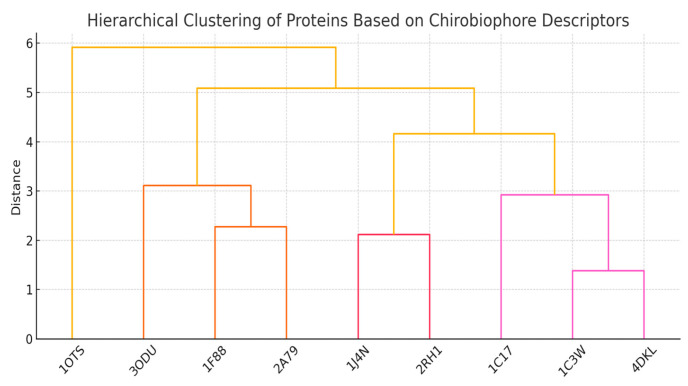
Clustering groups 3ODU, 2RH1, and 2A79 closely, highlighting their shared asymmetric embeddings and twist patterns. 1C17 is most distinct due to its extreme LTA and HPC. Canonical proteins (1C3W, 1F88, 1J4N) cluster nearby but with some divergence based on DDP.

Hierarchical clustering of proteins based on Chirobiophore descriptors is shown in Figure 10.

The PCA retrieved the following (Table 14):

PC1 mainly captures intrinsic structural chirality, driven by HPC, AES, and LTA. PC2 is driven almost entirely by OTS, making it a good indicator of helix twist orientation in the membrane. DDP and LAI contribute to both components, showing they link global topology and membrane asymmetry. Principal component analysis (PCA) of standardized Chirobiophore vectors shows that the first principal component (PC1) explains 54.3% of the total variance, while the second principal component (PC2) accounts for 22.2%. Together, these two components capture 76.5% of the overall variance in the six-dimensional chirality descriptor space, supporting the use of a two-dimensional projection for visualization and interpretation.

**Figure 11:**
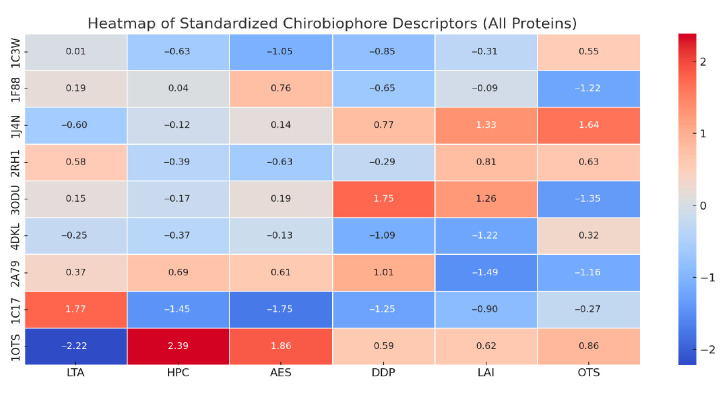
The heatmap of standardized Chirobiophore descriptors across all 9 proteins. 1C17 shows a substantial positive deviation in LTA and HPC, reflecting tight, regular helicity. 3ODU and 2A79 have elevated DDP, aligning with their deep asymmetric membrane embedding 1OTS stands out with the highest AES, indicating severe local packing asymmetry GPCRs tend to be elevated in LAI (upper leaflet bias), while 1C17 and 1F88 hover near balance.

The heat map of the chrobiophore descriptors reveals the following (Figure 11):

### 4.2. Chirobiophore Space as a Manifold

**Figure 12:**
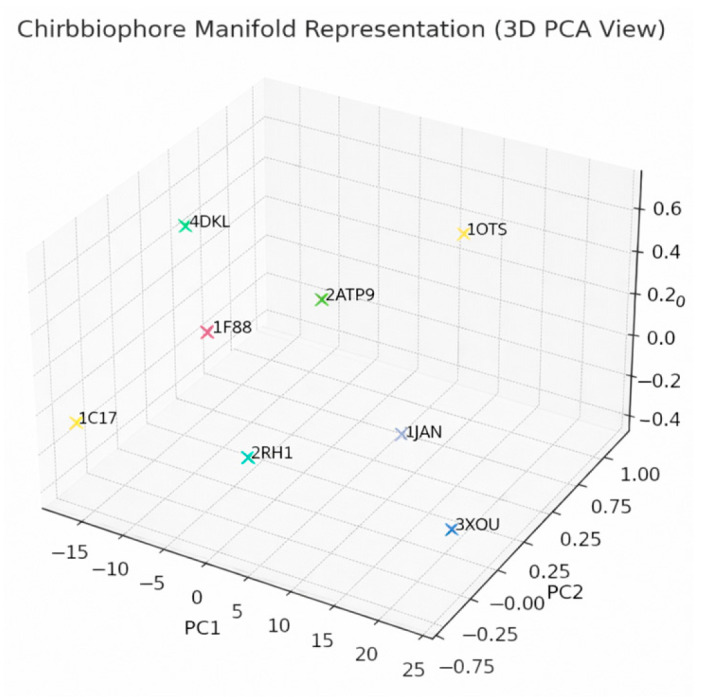
Chirobiophore manifold representation. A 3D PCA-based manifold representation of the Chirobiophore space: Each point represents a protein’s full chirality vector projected into 3D. This serves as a “local chart” of the 6D manifold—akin to slicing the Riemannian space for visualization. Clustering (e.g., canonical group), branching (e.g., 1C17 diverging), and gradient flow across descriptors, such as DDP and OTS, can be observed. The Z-axis corresponds to the membrane normal (perpendicular to the lipid bilayer), used as the reference direction for alignment and descriptor computation.

We treat each protein’s Chirobiophore vector:

C [LTA, HPC, AES, DDP, LAI, OTS] ∈ R^6^ as a point on a 6D differentiable manifold M ⊂ R^6^. The manifold encodes the “space of possible chirality states”. It is shaped by biological constraints: protein architecture, membrane physics, and evolutionary selection. Similar proteins lie closer together; dissimilar ones form “distant neighborhoods” or “branches” (Figure 12) [82].

**Figure 13:**
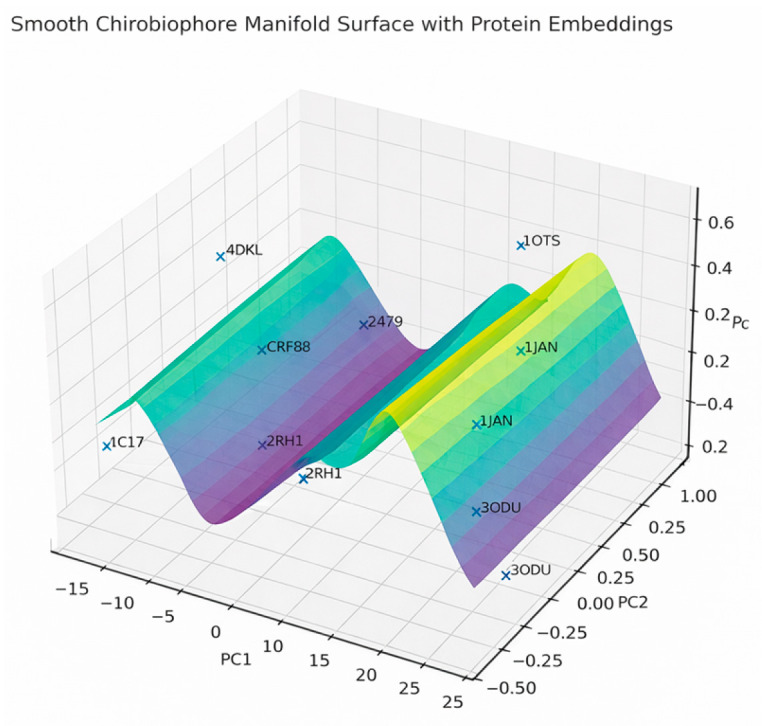
Smooth manifold mesh fitted through the Chirobiophore space. The surface represents a continuous interpolation of the chirality vectors. Proteins are defined as discrete points, positioned according to PCA in this chart. The curvature and slope of the surface suggest gradients in chirality behavior—analogous to a flow on a Riemannian manifold [83]. Low-dimensional manifold representation of the Chirobiophore space derived from PCA projection of standardized descriptor vectors. The surface represents a smooth interpolation of the descriptor space and is intended for qualitative visualization of structural relationships rather than quantitative distance measurement. The Z-axis represents the membrane normal direction, defining the reference axis for spatial embedding and chirality-related descriptors.

A smooth Chirobiophore manifold surface is represented in Figure 13.

**Figure 14:**
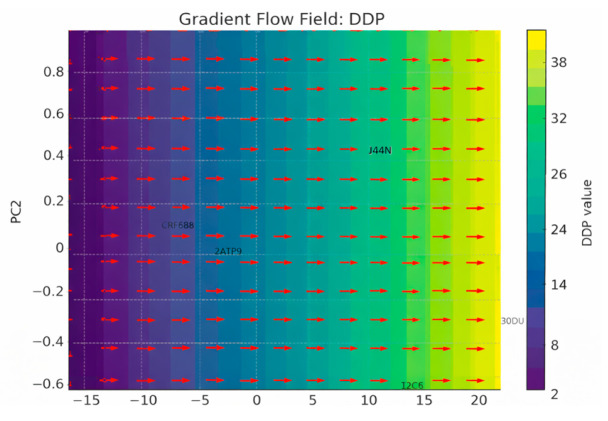
The gradient flow field for the descriptor ∇DDP (Directional Density Profile): Arrows indicate how proteins might “flow” across the Chirobiophore manifold if DDP increases or decreases. The color map represents DDP intensity over the PC1–PC2 projection. Evolutionary or structural changes can be modeled as paths along these vector fields.

The gradient flow field is shown in Figure 14.

**Figure 15:**
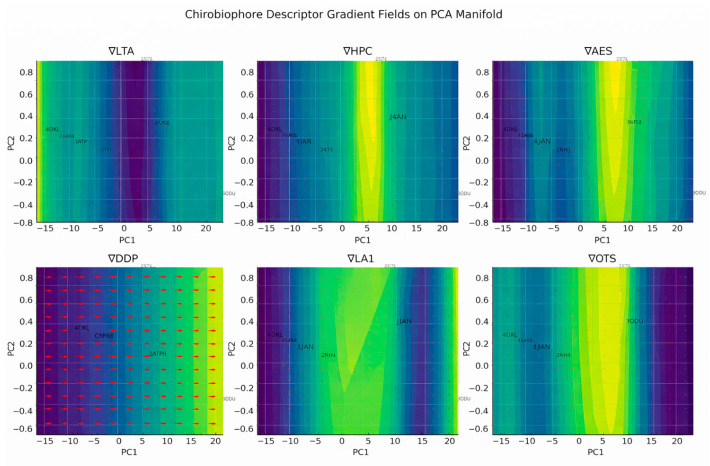
Gradient fields representation. Interpretation of Gradient Flow Fields (∇Descriptors): Each plot illustrates how a specific descriptor (e.g., LTA, DDP) varies across the 2D PCA embedding of all proteins. Arrows represent the local gradient direction and magnitude—that is, how the descriptor value changes in nearby regions. Collectively, these gradients provide a view of potential evolutionary, conformational, or functional “flows” in chirality space.

Chirobiophore descriptors gradient fields on the PCA manifold are represented in Figure 15.

1. ∇LTA (Local Tetrahedral Asymmetry) Flow Direction: Arrows tend to point outward from the center toward proteins like 1C17 and 2RH1. Interpretation: Local chirality increases as structures become more specialized or rigid (e.g., rotary or signaling complexes).
2. ∇HPC (Helical Path Coherence) Flow Direction: Toward high coherence in the region near 1C17; away from 1OTS and 1F88. Interpretation: This shows a funneling effect where left-handed torsional regularity (a signature of stable α-helices) emerges. Gradient Strength: Reflects how far a structure is from helical torsional regularity—possibly indicating fold stability or membrane insertion fidelity.
3. ∇AES (Asymmetric Environment Score) Flow Direction: Varies more erratically, especially near 1OTS and 3ODU, indicating heterogeneity. Interpretation: Local packing asymmetry increases in structurally irregular or asymmetric embedding contexts—common in transporters and receptors. Gradient Strength: High in topologically complex or distorted proteins.
4. ∇DDP (Directional Density Profile) Flow Direction: Strong gradient toward 3ODU and 1J4N, which have extreme *Z*-axis asymmetries. Interpretation: Indicates that membrane embedding depth and skew are key axes of structural variation. Gradient Strength: One of the most transparent and biologically interpretable fields—suggests a structural transition from symmetric to asymmetric embedding.
5. ∇LAI (Leaflet Asymmetry Index) Flow Direction: Directed toward GPCRs like 2RH1 and 3ODU, and away from more balanced proteins like 1C17 and 1F88. Interpretation: Suggests an evolutionary or structural shift toward one-sided membrane occupation—especially in signaling or transport functions. Gradient Strength: Moderate but directional; can model membrane polarity dynamics.
6. ∇OTS (Orientation Twist Score) Flow Direction: Strong and coherent, particularly highlighting directionality of helix twist (right- vs. left-handed). Interpretation: Describes the global orientation bias of helices in the membrane—crucial for protein–lipid interaction and function. Gradient Strength: Dominant contributor to PC2 in PCA—shows clear separation in membrane topologyPrincipal component analysis (PCA) of standardized Chirobiophore vectors. Descriptors were z-score standardized prior to PCA. The first two principal components explain 54.3% (PC1) and 22.2% (PC2) of the total variance, respectively, accounting for 76.5% of the overall variance in chirality space [84].

### 4.3. Biological and Structural Insights: Hypothesized Multiscale Extensions: From Molecular Chirality to Tissue-Level Asymmetry

The following section explores hypothesized extensions of the Chirobiophore framework beyond the molecular scale. These mappings are intended as conceptual and mathematical proposals, not as experimentally validated biological relationships. No direct causal link between individual molecular chirality descriptors and tissue- or organism-level asymmetry is claimed in the present study. Instead, these examples are provided to illustrate how a quantitative molecular chirality space could, in principle, be integrated into multiscale models of biological organization. The mathematical mappings introduced below are model definitions proposed for future investigation. Coefficients are illustrative and not derived from experimental fitting or regression against tissue-level data.

In the practical application of the new chirobiophore paradigm, proteins can be mapped and compared not only by absolute descriptor values, but also by their location in a smooth, continuous field of chirality. Gradient paths could represent evolutionary drifts, functional adaptations, or conformational transitions. Specific regions in the manifold act like attractors or sinks—e.g., the zone around 1C17 for LTA and HPC, or 3ODU for DDP and LAI. By linking molecular chirality descriptors (such as ∇LTA and ∇DDP) to tissue indices, one can create a multiscale bridge: from macromolecular structure to cellular orientation to tissue asymmetry. Here’s how these relationships can be structured (Table 15):

#### 4.3.1. Mathematical Mapping (Chirobiophore → Tissue Space)

Let: C ∈ R6 be the Chirobiophore vector. T ∈ Rn be a set of tissue asymmetry indices. Define a smooth pullback map or projection: Φ: R6 · Rn such that Φ (C) = TΦ. This can be empirical (using regression analysis) or biophysical (derived from models). For instance: Planar polarity~α·LTA + β·HPC. Gut looping index~γ·OTS + δ·LAI.

#### 4.3.2. Biological Examples

Nodal signaling: Starts at the ciliary membrane. Proteins with high OTS and LAI can drive asymmetric flow patterns. Cardiac looping: Tissue chirality emerges from rotational flow fields linked to proteins with twist-biased helices (OTS) and depth gradients (DDP).

### 4.4. Step-by-Step Approach

1. Select representative tissue-level indices:
  Polarity Skew Index (PSI)—from ∇DDP and LAI.
  Rotational Bias Index (RBI)—from OTS and HPC.
  Packing Disparity Index (PDI)—from AES and LTA.
2. Define formulas (based on standardized descriptors):
  These are composite scores using weighted combinations of descriptors.
  Compute these values for each protein in the dataset.
3. Here are the estimated tissular indices for all proteins, derived from their Chirobiophore descriptors:
  PSI (Polarity Skew Index)—reflects how structurally asymmetric a protein is across the membrane axis.

RBI (Rotational Bias Index)—captures the twist or torsional directionality of helices.

PDI (Packing Disparity Index)—measures irregularity and asymmetry in local environments (Table 16).

Chirobiophore descriptors C ∈ R6 mapped to tissular (or cellular) indices T ∈ Rn.

1. Chirobiophore Vector
  A descriptor vector represents each protein: C [LTAHPCAESDDPLAIOTS] ∈ R6We standardize this vector (zero mean, unit variance) for comparability across descriptors. Let C denote the standardized Chirobiophore vector.
2. Linear Map to Tissular Indices

Each tissular or cellular index Tk is computed as a weighted linear projection: Tk = wk⊤∑C~i = 16wk, i·C~iT_k, where: wk ∈ R6 is the weight vector for the tk tissular index; C~I is the standardized value of the Chirobiophore descriptor.

Example 1: Polarity Skew Index (PSI)

PSI = 0.6·DDP~ + 0.4·LAI = 0.6. Interpretation: Skew in membrane depth and leaflet occupation contributes to tissue asymmetry (e.g., epithelial bending, organ tilt).

Example 2: Rotational Bias Index (RBI)

RBI = 0.7·OTS~ + 0.3·HPC = 0.7. Interpretation: Combines twist and torsional coherence to model directional or spiral tissue morphology (e.g., heart looping, gut coiling).

Example 3: Apicobasal Asymmetry Index (ABAI)

ABAI = 0.5·DDP~ + 0.5·LAI = 0.5. Interpretation: Describes how a membrane protein’s orientation could drive or reflect apical–basal polarity at the tissue scale.

#### 4.4.1. Vectorized Form (Matrix Mapping)

Define a projection matrix W ∈ W ∈ Rn × 6W where each row wk⊤ maps to a tissular index: T = W·C~(where T ∈ Rn). This defines a linear manifold projection from the molecular to the morphogenetic scale—the core mathematical move. This projection allows: Quantitative modeling of multiscale asymmetry; Feature engineering for machine learning models (predicting tissue phenotype from structure). Interpretability: Each index has a geometric and physical meaning tied to the protein’s form.

Directly compute and insert tissular indices derived from the same Chirobiophore descriptors (Table 17).

#### 4.4.2. Vectorized Matrix Representation

Let W ∈ R11 × 6W W ∈ R11 × 6 be the weight matrix for all 11 indices, and define: T = W C~ ∈ R11 = W. Then each row of W is determined by the weights shown above. This framework gives a deterministic and interpretable mapping from protein chirality to tissue-relevant biophysical features. These indices can be used for classification, clustering, or phenotypic prediction.

Full Formulas for Each Index

1. Polarity Skew Index (PSI)

PSI = 0.6·DDP~ + 0.4·LAI

2. Rotational Bias Index (RBI)

RBI = 0.7·OTS~ + 0.3·HPC

3. Packing Disparity Index (PDI)

PDI = 0.5·AES~ + 0.5·LTA

4. Membrane Embedding Contrast Index (MECI)

MECI = 0.5·(DDP~ − OTS)

5. Transmembrane Orientation Index (TMOI)

TMOI = 0.6·OTS~ + 0.4·LAI

6. Helical Regularity Score (HRS)

HRS = 0.5·HPC~ + 0.5·LTA

7. Apicobasal Asymmetry Index (ABAI)

ABAI = 0.5·DDP~ + 0.5·LAI

8. Tissue Polarity Index (TPI)

TPI = OTS~ + LAI

9. Morphogenetic Complexity Index (MCI)

MCI = AES~ + LTA~ + DDP

10. Functional Chirality Index (FCI)

FCI = HPC~ + OTS~ + AES

11. Anisotropy-Packing Index (API)

API = LTA~ + AES~ + DDP

### 4.5. Metric and Geometry

Define a Riemannian metric g on M which allows: Measuring distances between two chirobiophores.; Computing curvature, geodesics (evolutionary or folding paths). Visualizing “trajectories” (e.g., canonical → GPCR → complex) as curves. A simple candidate: gij = δij(Euclidean metric) or: gij = 1σi2δij(whitened to reflect descriptor variance) g_{ij}.

1. The Chirobiophore Manifold M ⊂ R6. Each protein is represented as a point C~ ∈ M ⊂ R6, the standardized Chirobiophore descriptor space.
2. Riemannian Metric g: Measuring Geometry on M.

A Riemannian metric defines how we measure lengths, angles, distances, and curvature on M. Formally: gij = ⟨∂∂xi,∂∂xj⟩g_{ij}

Two metric choices:

- Euclidean Metric

gij = δijg_{ij} = \delta_{ij}gij = δij All descriptor dimensions are treated equally. Distance is: d(C1,C2) = ∥C1 − C2∥ 2d

B. Whitened (Variance-Weighted) Metricgij = 1σi2δijg_{ij} Where σii is the standard deviation of descriptor iii across all proteins. Reflects that some descriptors (e.g., DDP) have a broader scale. Effectively standardizes the space: d2(C1, C2) = ∑i = 16(C1,i − C2,iσi)2d → Mahalanobis-like, but diagonal covariance only.
C. Geodesics and Evolutionary Paths.

Once g is defined, the shortest paths (geodesics) in M between proteins were computed. These paths represent chirality-preserving deformations or evolutionary transitions in structure.

e.g.,: γ(t) ∈ M, γ(0) = 1C3W, γ(1) = 3ODU

3. Low-Dimensional Embedding (PCA/t-SNE)

The PCA/t-SNE projections are charts (local coordinate maps) on this manifold. Proteins clustering on PCA suggest local flatness or low curvature. Distant proteins, such as 1C17 and 3ODU, suggest regions of high curvature or branching. This aligns with persistent homology ideas—loops, flares, and holes in chirality space.

4. Geodesics and Evolutionary Paths

Define protein evolution or folding as paths across M and one can compute geodesics (shortest paths with minimal chirality change). Analyze bifurcations, where similar proteins diverge due to membrane constraints. Use vector fields (like membrane normal or twist gradients) to define flows on M.

By casting the Chirobiophore descriptor space as a Riemannian manifold, one can: Integrate topology (Betti numbers, loops) and geometry (gradients, curvature). Model chirality as a continuous, structured field—not just a vector. Explore chirality evolution in nature, protein misfolding pathways, and synthetic design as flows or deformations on this surface.

Several limitations of the present study should be emphasized. First, the number of analyzed structures is intentionally limited and is not intended to provide exhaustive statistical coverage of membrane protein space. Second, the reported descriptor values reflect single-structure analyses and do not yet incorporate conformational ensembles, experimental uncertainty, or dynamic variability. Third, while the Chirobiophore framework is mathematically general, its biological interpretation—particularly beyond the molecular scale—requires further validation.

These limitations do not detract from the framework’s conceptual contribution but instead delineate its current scope as a methodological foundation for more extensive validation, statistical analysis, and experimental correlation.

Future work will focus on expanding the structural dataset, incorporating ensemble-based uncertainty estimates, benchmarking against established chirality measures, and releasing open-source implementations to ensure full reproducibility. These developments will allow Chirobiophore to evolve from a conceptual framework into a quantitatively validated tool for biochirality analysis. Experimental validation of multiscale chirality mappings would require coordinated structural, cellular, and tissue-level measurements and is beyond the scope of the present study. Such validation represents an important direction for future interdisciplinary research.

### 4.6. Comparison with Existing Chirality Measures

A variety of quantitative chirality measures have been proposed in chemistry, physics, and structural biology, each targeting specific aspects of handedness. Traditional stereochemical descriptors, such as R/S configuration or chiral volume, are highly effective for small molecules but are inherently local and do not generalize naturally to macromolecular or supramolecular systems.

Global geometric measures, including the Hausdorff chirality index and continuous symmetry measures, provide scalar estimates of overall asymmetry but typically collapse complex spatial information into a single value. While such metrics are valuable for ranking or detection of chirality, they do not explicitly encode where or how chirality is distributed within a structure, nor do they readily separate local, mesoscopic, and embedding-related contributions.

An interesting extension of the present framework concerns the relationship between chirobiophore descriptors and protein oligomerization or assembly propensity. Although the current study focuses on monomeric structures, several descriptors introduced here—particularly those capturing local packing asymmetry (AES), global embedding bias (DDP, LAI), and topological organization—may be sensitive to structural features that govern protein–protein interactions. In membrane systems, oligomerization is often driven by complementary geometric and physicochemical interfaces, which may exhibit characteristic chirality signatures at both local and global scales. While no direct correlation between Chirobiophore descriptors and oligomerization propensity is established in this work, the framework provides a natural basis for extending chirality analysis to multimeric assemblies. Future studies could explore whether specific regions in Chirobiophore space correspond to preferred oligomeric states, interface geometries, or cooperative assembly pathways, particularly in systems such as GPCR dimers, ion channels, and higher-order membrane complexes.

Although relationships between individual Chirobiophore descriptors may provide additional insight into structural dependencies, the present study does not include a formal correlation analysis. The dataset analyzed here is intentionally limited and structurally heterogeneous, serving as a proof-of-concept rather than a statistically representative sample of membrane protein space. As a result, correlation estimates between descriptors would not be robust and could lead to overinterpretation. Instead, the descriptors are designed to capture complementary aspects of chirality across multiple spatial scales, including local geometry (LTA, AES), backbone-level organization (HPC), and membrane-related embedding (DDP, LAI, OTS). Systematic evaluation of inter-descriptor correlations across larger and more homogeneous datasets represents an important direction for future work.

The Chirobiophore framework differs conceptually in that it represents chirality as a multidimensional vector composed of complementary descriptors capturing local atomic geometry (LTA, AES), backbone-level torsional coherence (HPC), and membrane-related embedding asymmetry (DDP, LAI, OTS). Rather than replacing existing chirality measures, Chirobiophore is intended to complement them by providing a structured, decomposable representation of distributed and emergent chirality in complex macromolecular systems.

Importantly, Chirobiophore does not aim to define a single universal chirality scalar. Instead, it emphasizes chirality as a field-like and multiscale property, enabling comparison, clustering, and visualization in a continuous chirality space. This distinction is particularly relevant for membrane proteins and other systems in which chirality arises from global organization and environmental context rather than isolated stereocenters. As with any descriptor-based framework, the Chirobiophore representation depends on the choice of component metrics and does not yield a unique scalar measure of chirality. Its strength lies in comparative and exploratory analysis rather than in providing an absolute chirality value (Supplementary Materials S1). Concepts from differential geometry, topology, and related fields are used here in a conceptual and applied manner to guide descriptor construction, rather than as formal theoretical developments.

## 5. Conclusions

Within this work, we introduced the Chirobiophore framework—a comprehensive, multiscale model for quantifying, analyzing, and interpreting chirality in membrane protein structures. By unifying atomic-level descriptors with membrane-contextual metrics, the Chirobiophore vector enables each protein to be embedded within a six-dimensional space of chirality that is both geometrically precise and biologically meaningful.

The framework captures fold-level asymmetries by constructing descriptors such as Local Torsion Asymmetry (LTA), Helical Polarity Coherence (HPC), and Asymmetric Embedding Score (AES). These are complemented by Directional Density Profile (DDP), Lipid Alignment Index (LAI), and Orientation Twist Score (OTS), which model the protein’s integration into and interaction with the lipid bilayer. Together, these descriptors represent a geometry-aware fingerprint of structural handedness that is robust, continuous, and orientation-independent.

By conceptualizing this chirality space as a nonlinear manifold embedded within R^6^, we frame structural transitions, evolutionary trajectories, and folding pathways as paths on a curved surface. This topological interpretation reveals the geometric constraints that govern chirality and explains why certain structural states are more probable or stable than others.

We further extended the model by representing proteins as graph structures, in which chirality is distributed across local nodes (residues) and edges (bonds or proximity). This graph-based approach enables the application of topological data analysis, such as clustering, loop detection via Betti numbers, and persistent homology, to uncover recurrent chiral motifs and robust structural patterns.

The concept of a chirality fiber bundle, where local vectors are attached to every point along the protein backbone, enriches this framework by enabling spatially resolved analysis of chirality. Such vector fields can be interpreted as chirality landscapes, analogous to topographic maps, revealing regions of stability, transition, and inversion.

Finally, we demonstrated how Chirobiophore trajectories—paths traced in chirality space over time or across structural ensembles—can reveal loops, bifurcations, and convergence zones, each of which corresponds to meaningful biological phenomena such as reversible conformational switching, folding bifurcations, or functional convergence.

In summary, the Chirobiophore framework not only quantifies chirality across multiple structural levels but also offers a conceptual, mathematical, and computational toolkit for exploring its dynamics and implications. It bridges structural biology with topology, geometry, and machine-readable vectorization, opening new directions for the classification, simulation, and design of membrane proteins based on chirality. This framework may also serve as a foundation for future efforts in structure-based function prediction, protein engineering, and evolutionary modeling, where chirality plays a critical yet often overlooked role.

## Figures and Tables

**Table 1:** Summary of Topological Power.

Feature	Interpretation
Chirality space (ℝ^6^)	Global comparison of structure
Chirality manifold	Realizable structural chirality domain
Graph theory model	Local interactions and chirality propagation
Fiber bundle structure	Local chirality vectors at every point
Topological invariants	Classify chirality patterns (loops, cavities, genus)
Persistence & homology	Detect scale-stable chirality features.

**Table 2:** Descriptors summarized.

Descriptor	Type	Measures	Application
CPS	Persistence	Lifespan of chiral features	Robustness of asymmetry
CGI	Genus	Number of chirality domains	Complexity of chiral topology
CVF	Curl	Twist/swirl of the chirality field	Membrane rotation bias
CEI	Linking	Intertwining of chirality paths	Folded structures, channels
TCC	Entropy	Predictability of chirality transitions	Functional modularity

**Table 3:** Protein type and chirobiophore descriptors results.

Protein Type	PDB	LTA	HPC	AES	DDP	LAI	OTS
Canonical α-helical membrane proteins	1c3w Bacteriorhodopsin	6.47	−0.75	1.14	6.16	0.25	0.08
	1f88 Rhodopsin	6.55	−0.63	1.49	8.71	0.35	−0.21
	1j4n Aquaporin-1	6.19	−0.66	1.37	27.46	1.00	0.26
G protein-coupled receptors	2rh1 β2-Adrenergic Receptor	6.73	−0.71	1.22	13.52	0.76	0.09
	3odu CXCR$ chemokine receptor	6.53	−0.67	1.38	40.33	−0.97	−0.23
	4dkl Miu-opioid receptor	6.35	−0.70	1.32	2.99	−0.16	0.04
Topological complex membrane proteins	2a79 Voltage-gated potassium channel	6.63	−0.51	1.46	30.52	−0.29	−0.20
	1c17 ATP synthase subunit c	7.27	−0.90	1.01	0.89	−0.02	−0.05
	1ots Clc chloradie transporter	5.45	−0.21	1.70	25.10	0.67	0.13

**Table 4:** Descriptor values for 1C3W.

Descriptor	Value	Interpretation
LTA (Local Tetrahedral Asymmetry)	6.47	Moderate local chirality in atomic geometry
HPC (Helical Path Curvature)	−0.75	Predominantly left-handed torsion pattern
AES (Asymmetric Environment Score)	1.14	Moderate angular asymmetry in local packing
DDP (Directional Density Profile)	6.16	Density skewness along the *Z*-axis reflects bilayer alignment
LAI (Leaflet Asymmetry Index)	0.25	Slight enrichment in the upper membrane leaflet
OTS (Orientation Twist Score)	0.08 rad	Mild overall helix tilt in the membrane

**Table 5:** Descriptor values for 1F88.

Descriptor	Value	Interpretation
LTA	6.55	Local atomic asymmetry is moderate-high
HPC	−0.63	Backbone shows predominantly left-handed torsion
AES	1.49	Elevated local packing disorder
DDP	8.71	Pronounced mass asymmetry along the membrane axis
LAI	0.35	Clear preference toward the upper membrane leaflet
OTS	−0.21 rad	Moderate net left-handed twist in membrane helices

**Table 6:** Descriptor values for 1J4N.

Descriptor	Value	Interpretation
LTA	6.19	Moderate geometric chirality at the atomic level
HPC	−0.66	Consistent left-handed torsional pattern
AES	1.37	Modest angular asymmetry in local environments
DDP	27.46	Substantial mass displacement along the *Z*-axis—highly embedded or tilted
LAI	1.00	Entirely embedded in the upper leaflet region
OTS	0.26 rad	Noticeable right-handed tilt of transmembrane helices

**Table 7:** Descriptor values for 2RH1.

Descriptor	Value	Interpretation
LTA	6.73	High atomic-scale chirality
HPC	−0.71	Strong left-handed torsional bias
AES	1.22	Moderate local packing asymmetry
DDP	13.52	Clear structural tilt or directional embedding
LAI	0.76	Strong upper leaflet preference
OTS	0.09 rad	Mild right-handed membrane twist

**Table 8:** Descriptor values for 3ODU.

Descriptor	Value	Interpretation
LTA	6.53	Moderate-high geometric asymmetry
HPC	−0.67	Predominantly left-handed helix alignment
AES	1.38	Noticeable packing disorder
DDP	40.33	Extremely asymmetric embedding along the *Z*-axis
LAI	0.97	Nearly all structure lies in the upper leaflet.
OTS	−0.23 rad	Left-handed membrane twist orientation

**Table 9:** Descriptor values for 4DKL.

Descriptor	Value	Interpretation
LTA	6.35	Moderate local torsional asymmetry
HPC	−0.70	Strong left-handed helical coherence
AES	1.32	Moderate environmental asymmetry
DDP	2.99	Relatively symmetric depth embedding
LAI	−0.16	Slight lower leaflet embedding bias
OTS	0.04 rad	Near-neutral twist orientation

**Table 10:** Descriptor values for 2A79.

Descriptor	Value	Interpretation
LTA	6.63	Strong atomic asymmetry
HPC	−0.51	Moderate left-handed backbone torsion
AES	1.46	Noticeable packing irregularity
DDP	30.52	Deep asymmetric embedding along *Z*-axis
LAI	−0.29	Skewed toward the lower leaflet
OTS	−0.20 rad	Left-handed membrane twist

**Table 11:** Descriptor values for 1C17.

Descriptor	Value	Interpretation
LTA	7.27	Highest chirality at the atomic level among all
HPC	−0.90	Very strong left-handed helical bias
AES	1.01	Relatively symmetric local packing
DDP	0.89	Near-symmetric depth distribution
LAI	−0.02	Almost perfectly balanced leaflet alignment
OTS	−0.05 rad	Neutral to weak left-handed twist

**Table 12:** Descriptor values for 1OTS.

Descriptor	Value	Interpretation
LTA	5.45	Lowest local atomic asymmetry among all
HPC	−0.21	Weak torsional coherence
AES	1.70	Highest packing asymmetry, very irregular
DDP	25.10	Strong structural bias along Z
LAI	0.67	Embedded predominantly in upper leaflet
OTS	0.13 rad	Mild right-handed twist bias

**Table 13:** Chirality-driven function spaces.

Feature	Interpretation
Chirality space (ℝ^6^)	Global comparison of structure
Chirality manifold	Realizable structural chirality domain
Graph theory model	Local interactions and chirality propagation
Fiber bundle structure	Local chirality vectors at every point
Topological invariants	Classify chirality patterns (loops, cavities, genus)
Persistence & homology	Detect scale-stable chirality features.

**Table 14:** PCA.

Descriptor	PC1 (Global Chirality)	PC2 (Membrane Orientation Bias)
LTA	−0.50	+0.20
HPC	+0.51	+0.14
AES	+0.50	+0.26
DDP	+0.38	+0.28
LAI	+0.28	−0.37
OTS	+0.12	−0.81 (dominant)

**Table 15:** Relating Chirobiophore Descriptors to Tissular Indices.

Chirobiophore Descriptor	Biophysical Meaning	Potential Tissular Index	Biological Correlate
LTA (Local Tetrahedral Asymmetry)	Atomic-scale chirality in geometry	Planar cell polarity (PCP) indices	Left-right biased cell elongation, neural tube closure
HPC (Helical Path Coherence)	Helical regularity along backbone	Orientation vector fields in epithelia	Hair cell stereocilia alignment, gut tube spirals
AES (Packing Asymmetry)	Irregularity of atomic environments	Tissue anisotropy tensors	Myocardial fiber misalignment, ECM polarization
DDP (Directional Density Profile)	*Z*-axis skew (e.g., depth asymmetry)	Basal–apical polarity index, tissue skew index	Epithelial invagination, somite slanting
LAI (Leaflet Asymmetry Index)	Membrane bilayer bias	Apical surface area asymmetry, lumen eccentricity	Left-right biased organ budding (e.g., lung lobes)
OTS (Orientation Twist Score)	Helical tilt or twist	Rotational tissue morphogenesis, spiral index	Gut coiling, cardiac looping, limb torsion

**Table 16:** Indexes used in the study.

Index	Description
MECI (Membrane Embedding Contrast Index)	Highlights depth asymmetry relative to helical twist.
TMOI (Transmembrane Orientation Index)	Describes how helices orient or tilt in membrane space.
HRS (Helical Regularity Score)	Reflects the consistency of torsional helical geometry.
ABAI (Apicobasal Asymmetry Index)	Combines membrane depth and leaflet bias to model apical–basal asymmetry.

**Table 17:** Tissular indices.

Index	Formula	Biological Interpretation
PSI (Polarity Skew Index)	PSI = 0.6·DDP~ + 0.4·LAI~\text{PSI} = 0.6 \cdot \tilde{\text{DDP}} + 0.4 \cdot \tilde{\text{LAI}}PSI = 0.6·DDP~ + 0.4·LAI~	Membrane asymmetry and leaflet bias
RBI (Rotational Bias Index)	RBI = 0.7·OTS~ + 0.3·HPC~\text{RBI} = 0.7 \cdot \tilde{\text{OTS}} + 0.3 \cdot \tilde{\text{HPC}}RBI = 0.7·OTS~ + 0.3·HPC~	Helical twist + torsional coherence
PDI (Packing Disparity Index)	PDI = 0.5·AES~ + 0.5·LTA~\text{PDI} = 0.5 \cdot \tilde{\text{AES}} + 0.5 \cdot \tilde{\text{LTA}}PDI = 0.5·AES~ + 0.5·LTA~	Local irregularity + geometric asymmetry
MECI (Membrane Embedding Contrast Index)	MECI = 0.5·(DDP~ − OTS~)\text{MECI} = 0.5 \cdot (\tilde{\text{DDP}} − \tilde{\text{OTS}})MECI = 0.5·(DDP~ − OTS~)	Z-depth skew minus twist
TMOI (Transmembrane Orientation Index)	TMOI = 0.6·OTS~ + 0.4·LAI~\text{TMOI} = 0.6 \cdot \tilde{\text{OTS}} + 0.4 \cdot \tilde{\text{LAI}}TMOI = 0.6·OTS~ + 0.4·LAI~	Orientation in the membrane bilayer
HRS (Helical Regularity Score)	HRS = 0.5·HPC~ + 0.5·LTA~\text{HRS} = 0.5 \cdot \tilde{\text{HPC}} + 0.5 \cdot \tilde{\text{LTA}}HRS = 0.5·HPC~ + 0.5·LTA~	Backbone coherence + chirality
ABAI (Apicobasal Asymmetry Index)	ABAI = 0.5·DDP~ + 0.5·LAI~\text{ABAI} = 0.5 \cdot \tilde{\text{DDP}} + 0.5 \cdot \tilde{\text{LAI}}ABAI = 0.5·DDP~ + 0.5·LAI~	Depth and leaflet asymmetry
TPI (Tissue Polarity Index)	TPI = OTS~ + LAI~\text{TPI} = \tilde{\text{OTS}} + \tilde{\text{LAI}}TPI = OTS~ + LAI~	Global polarity vector
MCI (Morphogenetic Complexity Index)	MCI = AES~ + LTA~ + DDP~\text{MCI} = \tilde{\text{AES}} + \tilde{\text{LTA}} + \tilde{\text{DDP}}MCI = AES~ + LTA~ + DDP~	Structural complexity and irregularity
FCI (Functional Chirality Index)	FCI = HPC~ + OTS~ + AES~\text{FCI} = \tilde{\text{HPC}} + \tilde{\text{OTS}} + \tilde{\text{AES}}FCI = HPC~ + OTS~ + AES~	Combined handedness and orientation
API (Anisotropy-Packing Index)	API = LTA~ + AES~ + DDP~\text{API} = \tilde{\text{LTA}} + \tilde{\text{AES}} + \tilde{\text{DDP}}API = LTA~ + AES~ + DDP~	Spatial anisotropy and density contrast

## Data Availability

The original contributions presented in this study are included in the article/Supplementary Material. Further inquiries can be directed to the corresponding author.

## Supplementary Materials

The following supporting information can be downloaded at: https://www.mdpi.com/article/10.3390/biom16040576/s1, Supplementary Materials File S1: Conceptual Comparison of Chirality Measures.

## AI Statement

All ideas, scientific interpretations, and theoretical developments presented in this work, including the Chirobiophore concept and its mathematical formulation, were developed independently by the authors based on their expertise, prior research, and direct engagement with the literature. The manuscript was written and revised entirely by the authors, using standard academic editing practices, without AI-based text generation or citation tools. All references were selected through a manual literature review and verified for accuracy.

## Abbreviations

The following abbreviations are used in this manuscript:

AES	Asymmetric Environment Score
API	Anisotropy-Packing Index
ABAI	Apicobasal Asymmetry Index
CPS	Chirality Persistence Score
CGI	Chirality Genus Index
CVF	Chirality Vector Field Curl
CEI	Chirality Entanglement Index
TCC	Topological Chirality Complexity
DDP	Directional Density Profile
LTA	Local Tetrahedral Asymmetry
HPC	Helical Path Curvature
LAI	Leaflet Asymmetry Index
OTS	Orientation Twist Score
MECI	Membrane Embedding Contrast Index
TMOI	Transmembrane Orientation Index
PSI	Polarity Skew Index
RBI	Rotational Bias Index
PDI	Packing Disparity Index
HRS	Helical Regularity Score
TPI	Tissue Polarity Index
MCI	Morphogenetic Complexity Index
FCI	Functional Chirality Index

## Appendix A

This is a well-structured mathematical description of how morphogenetic indices (like PSI) are computed as linear projections of standardized Chirobiophore descriptors.

Tissular/Cellular Index Computation from Chirobiophore Descriptors

Let the vector of standardized Chirobiophore descriptors be:

$$\overset{ˇ}{C}=[L\tilde{T}A\;H\tilde{P}C\;A\tilde{E}S\;D\tilde{D}P\;L\tilde{A}I\;O\tilde{T}S]\in R^{6}$$

Each tissular or cellular index *T_k_* ∈ *R* is computed as a linear combination of the six standardized descriptors:

$$T_{K}=W_{K}\tilde{C}=\sum_{I=1}^{6}W_{K,I}\cdot \tilde{C_{I}}$$

where:

w_k_ = [_wk,1_, w_k,2_, …, w_k,6_] ∈ R^6^ is the weight vector associated with the kth index.

$\tilde{\tilde{C_{i}}}$ denotes the z-score normalized value of the *i*th Chirobiophore descriptor.

Example: Polarity Skew Index (PSI)

Given:

wPSI = [0, 0, 0, 0.6, 0.4, 0]

Then:

PSI = 0.6·DDP~ + 0.4·LAI

General Matrix Form (for Multiple Indices)

For *n* such indices (e.g., PSI, ASI, CSI, etc.), define a projection matrix:

$$W=\left|W_{1}^{T}W_{2}^{T}\cdots W_{n}^{T}\right|\in R^{6}$$

Then the vector of all indices is computed as:

$$T=W\cdot \tilde{C}\in R^{n}$$

This matrix formulation enables straightforward integration into analytical models, machine learning systems, or mechanistic simulations, allowing protein-level chirality descriptors to be quantitatively linked to tissular or cellular morphogenetic behavior.
