# Supplementary material for: Chirobiophore: A Novel Framework for Quantifying Biochirality in Macromolecular Systems
Source: Biomolecules. 2026 Apr 13;16(4):576. doi: 10.3390/biom16040576 (PMC13113911; doi:10.3390/biom16040576)
Supplement: Supplementary file 1 [file biomolecules-16-00576-s001.zip › biomolecules-4205810-Supplementary Materials File S1.pdf]

Supplementary material

Supplementary Materials File S1 Conceptual Comparison of Chirality Measures

Approach	Scale	Output	Strengths	Limitations
**Chiral volume / R–S**	Local	Scalar	Precise stereochemistry	Not applicable to macromolecules
**Hausdorff chirality**	Global	Scalar	Continuous, orientation-invariant	No spatial decomposition
**Continuous symmetry measures**	Global	Scalar	Quantifies deviation from symmetry	Limited interpretability
**Chirobiophore (this work)**	Multiscale	Vector	Distributed, decomposable, context-aware	Requires multiple descriptors; not a single scalar
